# Effective mathematical modelling of health passes during a pandemic

**DOI:** 10.1038/s41598-022-10663-5

**Published:** 2022-04-28

**Authors:** Stefan Hohenegger, Giacomo Cacciapaglia, Francesco Sannino

**Affiliations:** 1 Institut de Physique des 2 Infinis (IP2I) de Lyon, CNRS/IN2P3, UMR5822, 69622 Villeurbanne, France; 2 Université de Lyon, Université Claude Bernard Lyon 1, 69001 Lyon, France; 3 Scuola Superiore Meridionale, Largo S. Marcellino, 10, 80138 Naples, NA Italy; 4 Dipartimento di Fisica, E. Pancini, Università di Napoli, Federico II and INFN sezione di Napoli, Complesso Universitario di Monte S. Angelo Edificio 6, via Cintia, 80126 Naples, Italy; 5 CP3-Origins and D-IAS, University of Southern Denmark, Campusvej 55, 5230 Odense, Denmark

**Keywords:** Health policy, Applied mathematics

## Abstract

We study the impact on the epidemiological dynamics of a class of restrictive measures that are aimed at reducing the number of contacts of individuals who have a higher risk of being infected with a transmittable disease. Such measures are currently either implemented or at least discussed in numerous countries worldwide to ward off a potential new wave of COVID-19. They come in the form of Health Passes (HP), which grant full access to public life only to individuals with a certificate that proves that they have either been fully vaccinated, have recovered from a previous infection or have recently tested negative to SARS-Cov-2. We develop both a compartmental model as well as an epidemic Renormalisation Group approach, which is capable of describing the dynamics over a longer period of time, notably an entire epidemiological wave. Introducing different versions of HPs in this model, we are capable of providing quantitative estimates on the effectiveness of the underlying measures as a function of the fraction of the population that is vaccinated and the vaccination rate. We apply our models to the latest COVID-19 wave in several European countries, notably Germany and Austria, which validate our theoretical findings.

## Introduction

The epidemiological dynamics of SARS-Cov-2  in many countries has been characterised by several waves. These are periods of exponential growth in the number of infected individuals, followed by (quasi-)linear growth phases. Modelling this dynamics in 2021 is involved due to a number of different factors: (1) the availability of several different vaccines, which started being deployed at the end of 2020, and national vaccination campaigns around the globe; (2) the appearance of several variants of SARS-Cov-2 , which differ in their infection rate^1–4^ and their ability to avoid antibody responses (recent theoretical^5^ and numerical^6^ studies explore the impact of variants on the pandemic diffusion); (3) non-pharmaceutical interventions (ranging from lockdowns to various degrees of social distancing measures) taking into account economical, social and political factors. In particular regarding i), roughly $37\%$ of the global population is fully vaccinated as of the end of October 2021, see Ourworldindata.org, however with only very few countries having reached a rate of $>50\%$, which is still largely below the projected herd-immunity threshold.

With the number of vaccinated adult individuals rising (but still staying below the herd immunity threshold, in particular for the more aggressive new Delta-variant) and in an attempt to further allow social life to return to levels similar to the ones before the pandemic, many countries have discussed (and in several cases also adopted) social distancing measures that are tailored according to the threat an individual poses to infect others. Such measures require individuals to present certificates, which prove that they present a low risk of being infectious, in order to participate in the public life. The certificates attest that the person is fully vaccinated against COVID-19 (after having received the required number of doses of an approved vaccine and a certain waiting time), or that they have recovered from a not too distant infection or that they have recently tested negative for SARS-Cov-2. In fact, different combinations of the above are present at national level. The social measure requires to present the certificate before entering locations or events where the risk of contagion is high^7^, such as public places (restaurants, bars, museums, shopping malls etc.), social events (concerts, theatres, cinemas etc.), public means of transportation (trains, airplanes,etc.) or universities and schools. Since the specifics and the names differ from country to country, in the following we shall collectively refer to these certificates as *Health Passes* (HPs). Examples for concrete implementations in different European countries can be found in Section S6.1 of the Supplementary Material.

The objective of our work is to develop a simple and economical mathematical model that allows to analyse the impact of different versions of HPs on the epidemiological dynamics of an entire wave of a pandemic. The mathematical modelling of infectious diseases has a long standing history, and several different approaches exist in the literature: these range from stochastic methods^8–13^ to deterministic compartmental models^14–16^ (including the effect of vaccines^17–20^) to computer- and data driven approaches^21–25^ and network models^26–28^. The advent of SARS-Cov-2  has caused a spike of research activity, with new models exploring key aspects of the pandemic, for example its multi-wave structure^29,30^ and the evaluation of its impact on society and economy^31–36^. Models that quantify the impact of non-pharmaceutical interventions have also been proposed^37–40^ and validated on the COVID-19 available data. Nevertheless, the specific impact of HPs has not been studied in the literature yet, and our work aims at covering this gap. In the current work we shall exploit the interplay between two types of models, namely a *compartmental model* and the *epidemiological Renormalisation Group* (eRG) approach. The former is among the oldest approaches^14^ (see^16,41^ for further references) and it treats the spread of an infectious disease by dividing the population into several compartments containing individuals in different states with respect to the disease (e.g. susceptible, exposed, infectious, recovered etc.). The passage of individuals from one compartment to another is described through a set of coupled first order differential equations in time, which can be seen as a continuous mean-field approximations of a more microscopic description of the infections^12,13,41^. Models of this type can be easily adapted and extended^31^ by adding compartments^42,43^, stratifying them in terms of age groups or geographical location^44^ and upgrading the parameter to functions of time for a better fit to the data. Compartmental models are particularly useful in establishing qualitative relations between microscopic aspects of the spread of the disease among individuals and more macroscopic observables, such as the total number of individuals who have become infected at the end of an epidemic wave. However, in their simplest incarnation with constant transition rates, these models are capable of describing the time evolution accurately only over a relatively short period of time. Due to the fact that the epidemiological situation constantly changes (as we explained above), the rates need to be adapted as functions of time. The eRG framework has been introduced^29,30^ to capture, more efficiently, the time evolution of the disease diffusion by explicitly taking into account symmetry, being inspired by the physical concepts of time-invariance symmetry and fixed points (the concept of Renormalisation Group Equations (RGE) has been originally developed in the context of statistical and particle physics^45–47^). Concretely, the eRG framework takes the form of a set of flow equations (called the $\beta$-functions) that describe the evolution of a quantity of epidemiological interest (e.g. a smooth monotonic function of the cumulative number of infected individuals $I_{\text {c}}$) as the flow between different fixed points. It has been demonstrated^48,49^ that the eRG approach is indeed capable of describing accurately not only a full wave of COVID-19, but is also capable of modelling more complex multi-wave structures^50,51^ even under changing conditions, like the appearance of new variants^5^, vaccination dynamics^52^, change in the social dynamics^53^, among the ones mentioned above.

**Figure 1:**
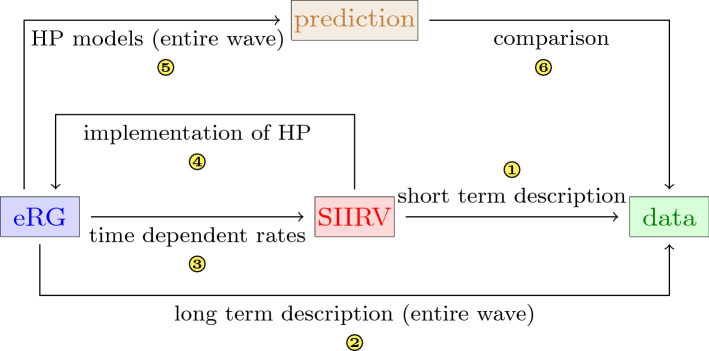
Schematic overview of the interplay between a compartmental model (SIIRV) and an eRG approach to arrive at quantitive prediction on HP measures from input data.

In this work we studied the effectiveness of HPs by combining the flexibility of compartmental models in capturing microscopic details of the spread of a disease and their relation to more macroscopic quantities within an eRG framework. The latter efficiently encapsulates the symmetries and long-term aspects of epidemics. The methodology we followed is schematically illustrated in Fig. 1: we first introduce a compartmental model (called SIIRV) which contains 5 compartments, namely $S$ (unvaccinated susceptible), $I_1$ (unvaccinated infectious), $I_2$ (previously vaccinated infectious), $R$ (removed) and $V$ (vaccinated susceptible). Although vaccines have fairly high efficacies in preventing infections in the vaccinated population (around 80–90% for the best cases), here we take into account the fact that vaccines neither grant complete immunity against an infection from SARS-Cov-2  nor prevent the transmission of the virus from an infected vaccinated individuals to others. Assuming constant rates at which individuals pass between these compartments, this model is capable of fitting epidemiological data only over a short period of time ①. Alas, this class of models is not capable of describing correctly the dynamics of an entire wave of COVID-19. Hence, we ensure a correct description of the epidemiological data via the eRG framework ②. Matching the solutions of the SIIRV model with the eRG provides time-dependent infection and removal rates ③, which allow to extend the range of validity of the compartmental model to accurately reproduce the data. We then implement two different types of HP-models at the level of the SIIRV model (with time-dependent rates): the net effect is a reduction in the contact rates between certain classes of individuals, thus corresponding to a systematic re-scaling of certain terms in the differential equations controlled by an efficacy parameter *p*. Studying the dynamics of the resulting system allows us to implement the HP-models in the eRG ④ by introducing a *p*-dependence in its parameters. This provides us with a model that not only allows to make long-term predictions ⑤ for HP-models but also to compare ⑥ different types of HP  among each other and the current situation in different countries.

We consider two different types of HP-measures putting different emphasis on vaccinated versus unvaccinated individuals:

- Vaccine and Test Health Passes (VT-HP): individuals with a certificate of a negative test against SARS-Cov-2  are granted the same level of access to public life as people who have been vaccinated. We implicitly assume, in this scenario, that tests are easily accessible (and free of charge) for the majority of the population. Examples for models of this type which have actually been implemented are the Austrian ‘3-G-Regel’, the Danish ‘Corona Pass’ or the French ‘pass sanitaire’ .
- Vaccine Health Passes (V-HP): only individuals who posses a certificate for being completely vaccinated against SARS-Cov-2  are granted unrestricted access to public life

In both cases, individuals that have previously contracted the disease are considered as fully immunised. Currently, there are several examples of VT-HPs implemented in various countries, while (to our knowledge) V-HPs are currently only being discussed. We consider the two HP  models as templates of two extremal situations, and perform a comparative analysis of their effect on the long-term spread of the disease.

## Methods

### Compartmental vaccine model and health passes

We first introduce a compartmental model, conceived to describe and quantify the impact of HPs during a single epidemiological wave. For COVID-19, this requires a typical time-span of 1–2 months, hence variations in the population due to births, mobility and mortality play a minor role. Hence, the model we design is based on the following main assumptions:

1. A closed population, comprising of all the individuals that are susceptible of being infected during a single epidemic wave.
2. Negligible reinfection rates, hence all individuals that are unable to infect (deceased, recovered, quarantined, hospitalised individuals) are counted in a single compartment.
3. Merged categories when they are not distinguished by the application of the Health Pass: for simplicity, we do not consider deaths, hospitalisations, isolation, etc, as separate compartments. This greatly reduces the number of free parameters and improves on predictivity.
4. Separated compartments for vaccinated and un-vaccinated individuals.
5. Time-independent parameters (infection, recovery and vaccination rates).

This leads to the 5-compartment model, which we describe in detail below.

#### Basic SIIRV model

Our starting point is an isolated population of size $N\gg 1$, which we re-group into 5 basic compartments, as listed below.

- Susceptible: $N\,S(t)$ denotes the number individuals at time *t* who are not infectious and who have not been (fully) vaccinated. They can become infectious if they come in contact with the disease via an infectious individual.
- Vaccinated: $N\,V(t)$ denotes the number of individuals who, at time *t*, are not infectious and who are fully vaccinated. We shall, however, assume that these individuals can still get infected if they come in contact with the disease, but with a rate suppressed by a factor $\zeta < 1$ compared to $S$.
- Infectious: $N\,I_1(t)$ denotes the number of infectious individuals at time *t* who have not been previously vaccinated.
- Infectious: $N\,I_2(t)$ denotes the number of infectious individuals at time *t* who have been previously vaccinated.
- Removed: $N\,R(t)$ denotes the number of removed individuals at time *t*, who cannot become infectious. They account for previously infectious individuals who fully recovered, or are prevented from infecting due to some other removal mechanism (such as quarantine or death).

Individuals can pass from one of these compartments to another through various mechanisms, which we model through fixed rates $\gamma _{1,2}$ (infection), $\varepsilon$ (removal) and $\rho$ (vaccination rate). The processes are mathematically described by the following coupled first order differential equations in time:

1 $$\begin{aligned} \frac{dS}{dt}(t)&=-S(t)\,\left[ \rho +\gamma _1\,I_1(t)+\gamma _2\,I_2(t)\right],&\frac{dI_1}{dt}(t)=S(t)\,\left[ \gamma _1\,I_1(t)+\gamma _2\,I_2(t)\right] -\varepsilon \,I_1(t),\nonumber \\ \frac{dV}{dt}(t)&=\rho \,S(t)-V(t)\,\zeta \,\left[ \gamma _1\,I_1(t)+\gamma _2\,I_2(t)\right] \,,&\frac{dI_2}{dt}(t)=V(t)\,\zeta \,\left[ \gamma _1\,I_1(t)+\gamma _2\,I_2(t)\right] -\varepsilon \,I_2(t),\nonumber \\ \frac{dR}{dt}(t)&=\varepsilon \,\left[ I_1(t)+I_2(t)\right] , \end{aligned}$$

which need to be supplemented by the initial conditions

2 $$\begin{aligned}&S(t=0)=S_0\,,&I_1(t=0)=I_{1,0}\,,&I_2(t=0)=I_{2,0}\,,&V(t=0)=V_0\,,&R(t=0)=0\,. \end{aligned}$$

Here we assume the outbreak of the disease at $t=0$ and we normalise the initial conditions to satisfy $S_0+I_{1,0}+I_{2,0}+V_0=1$. In (1), $\gamma _1,\gamma _2 \in {\mathbb {R}}_+$ are the rates at which infectious individuals with or without prior vaccination infect susceptible individuals. These two rates are not considered a priori the same (however, in most examples, for simplicity we used $\gamma _1=\gamma _2$). Furthermore, $\varepsilon$ denotes the recovery rate, which is assumed to be independent of whether individuals have been previously vaccinated or not. We also define the ratios $\sigma _1=\frac{\gamma _1}{\varepsilon }$ and $\sigma _2=\frac{\gamma _2}{\varepsilon }$, which correspond to the reproduction numbers of the two infectious compartments. The rate $\rho$ in (1) denotes the vaccination rate, which is chosen to be constant. Studying the examples of Germany and Austria, evidence is provided in Sections S6.2 and S6.3 of the Supplementary Material that this indeed leads to a reasonable approximation for a single wave of COVID-19. Finally, the efficacy of the vaccine is encoded in the reduction factor $\zeta \in [0,1)$ for the infection rate of vaccinated individuals, estimated from recent studies for different vaccines agains SARS-Cov-2^54^. For later use, we also define the cumulative number of infected individuals as a function of time:

3 $$\begin{aligned} I_{\text {c}}(t)&=N\,(I_{1,0}+I_{2,0})+N\int _0^t dt'\,\left[ S(t')+\zeta \,V(t')\right] \,\left[ \gamma _1\,I_1(t')+\gamma _2\,I_2(t')\right] \,. \end{aligned}$$

Finally, the herd-immunity threshold for the vaccination dynamics encoded in eqs. (1) is $h^{\text {HIT}}=\frac{\sigma _1-1}{\sigma _1-\zeta \sigma _2}$ (see Section S1.2 of the Supplementary Material for more details).

#### Implementing health passes

The factor $\zeta$ in the SIIRV model (1) mainly takes into account biological effects of the various vaccines and a priori is not related to any social distancing measures particularly targeted at unvaccinated individuals. HP-measures, instead, are specifically aimed at reducing social contacts of individuals posing a higher threat of infecting others by allowing access to public places and social events only to individuals who can either prove a certain level of immunisation against SARS-Cov-2  and/or have recently tested negative for the virus. To implement these measures into the SIIRV model, we distinguish the two conceptually different types of HPs, as described in the introduction:

- VT-HP: this model effectively only restricts the contacts of unvaccinated infectious individuals, i.e. those in the compartment $I_1$. In (1), we can implement such restrictions through a suppression factor $p_{\text {VT}}\in [0,1]$ that takes into account how much contacts of the unvaccinated infectious individuals $I_1$ with the rest of the population are reduced
  4 $$\begin{aligned} \frac{dS}{dt}&=-S\,\left[ \rho +p_{\text {VT}}\,\gamma _1\,I_1(t)+\gamma _2\,I_2\right] \,,\nonumber \\ \frac{dI_1}{dt}&=S\,\left[ p_{\text {VT}}\,\gamma _1\,I_1+\gamma _2\,I_2\right] -\varepsilon \,I_1\,,\nonumber \\ \frac{dV}{dt}&=\rho \,S-V\,\zeta \,\left[ p_{\text {VT}}\,\gamma _1\,I_1+\gamma _2\,I_2\right] \,,\nonumber \\ \frac{dI_2}{dt}&=V\,\zeta \,\left[ p_{\text {VT}}\,\gamma _1\,I_1+\gamma _2\,I_2\right] -\varepsilon \,I_2\,,\nonumber \\ \frac{dR}{dt}&=\varepsilon \,\left[ I_1+I_2\right] \,. \end{aligned}$$
  Mathematically, the VT-HPcorresponds to a rescaling of the infection rate for unvaccinated individuals $\gamma _1 \rightarrow p_{\text {VT}}\gamma _1$. The cumulative number of infected individuals for this model becomes
  5 $$\begin{aligned} I^{(\text {VT})}_{\text {c}}(t,p_{\text {VT}})&=N\,(I_{1,0}+I_{2,0})+N\int _0^t dt'\,\left[ S(t')+\zeta \,V(t')\right] \,\left[ p_{\text {VT}}\,\gamma _1\,I_1(t')+\gamma _2\,I_2(t')\right] \,. \end{aligned}$$
- V-HP: in models of this type, the social interactions of any unvaccinated individual (i.e. in the compartments $S$ and $I_1$) are reduced. In (1), we can implement such restrictions through a suppression factor $p_{\text {V}}\in [0,1]$ that measures the efficacy of this reduction
  6 $$\begin{aligned} \frac{dS}{dt}&=-S\,\left[ \rho +p_{\text {V}}^2\,\gamma _1\,I_1+p_{\text {V}}\,\gamma _2\,I_2\right] \,,\nonumber \\ \frac{dI_1}{dt}&=S\,\left[ p_{\text {V}}^2\,\gamma _1\,I_1+p_{\text {V}}\,\gamma _2\,I_2\right] -\varepsilon \,I_1\,,\nonumber \\ \frac{dV}{dt}&=\rho \,S-V\,\zeta \,\left[ p_{\text {V}}\,\gamma _1\,I_1+\gamma _2\,I_2\right] \,,\nonumber \\ \frac{dI_2}{dt}&=V\,\zeta \,\left[ p_{\text {V}}\,\gamma _1\,I_1+\gamma _2\,I_2\right] -\varepsilon \,I_2\,,\nonumber \\ \frac{dR}{dt}&=\varepsilon \,\left[ I_1+I_2\right] \,. \end{aligned}$$
  The cumulative number of infected individuals is given by
  7 $$\begin{aligned} I^{(\text {V})}_{\text {c}}(t,p_{\text {V}})&=N\,(I_{1,0}+I_{2,0})+N\int _0^t dt'\,\left[ p_{\text {V}}\,S(t')+\zeta \,V(t')\right] \,\left[ p_{\text {V}}\,\gamma _1\,I_1(t')+\gamma _2\,I_2(t')\right] \,. \end{aligned}$$

### Epidemiological renormalisation group

Here we use the simplest eRG approach^29^, which follows the time-evolution of the number of infections in a closed and isolated population over the time-span of a single wave. This formulation is enough to study the effect of HPs. In the literature, extensions of the eRG are available, which include the multi-wave pattern^50,51^, people’s mobility between different regions^48,49,52^ and the presence of multiple virus variants^5^.

#### Time dependent rates and relation to SIIRV

The eRG approach^29,30^ describes the spread of a disease through flow equations (the so called $\beta$-functions) and characterises a wave as the flow between fixed points^5,29,49,50^. Concretely, for $\alpha =\phi (I_{\text {c}})$, with $\phi$ a continuous, differentiable and monotonic function, the $\beta$-function for a single wave (and a single variant of a disease) can be written as

8 $$\begin{aligned} -\beta _{\alpha }(t)=\frac{d\alpha }{dt}=\frac{d\phi }{dI_{\text {c}}}\,\frac{dI_{\text {c}}}{dt}=\lambda _0\,\alpha \,\left( 1-\frac{\alpha }{A_0}\right) ^{2d}\,, \end{aligned}$$

with $(A_0,\lambda _0,d)$ constants. Here, $\lambda _0$ is related to the infection rate of the disease, while $A_0$ is the asymptotic number of individuals who get infected during the wave. For simplicity, we shall consider $d=\frac{1}{2}$ and $\alpha =\phi (I_{\text {c}})=I_{\text {c}}$ in the following. In this case, the solution of the flow equation (8) is a logistic function

9 $$\begin{aligned} I_{\text {c}}(t)=\frac{A_0}{1+e^{-\lambda _0(t-t_0)}}\,, \end{aligned}$$

where $t_0\in {\mathbb {R}}$ is an integration constant that gives the timing of the maximum of infectious individuals during the wave. As demonstrated in the literature^5,29,30,48,49^, and shown in more examples in Section S6 of the Supplementary Material, for suitable values of the parameters $(A_0,\lambda _0,t_0)$, the function (9) describes accurately the time evolution of infected individuals during a single wave of COVID-19 even for populations that differ greatly geographically as well as socio-culturally and under very different circumstances regarding non-pharmaceutical interventions, vaccines and variants of SARS-Cov-2 .

**Figure 2:**
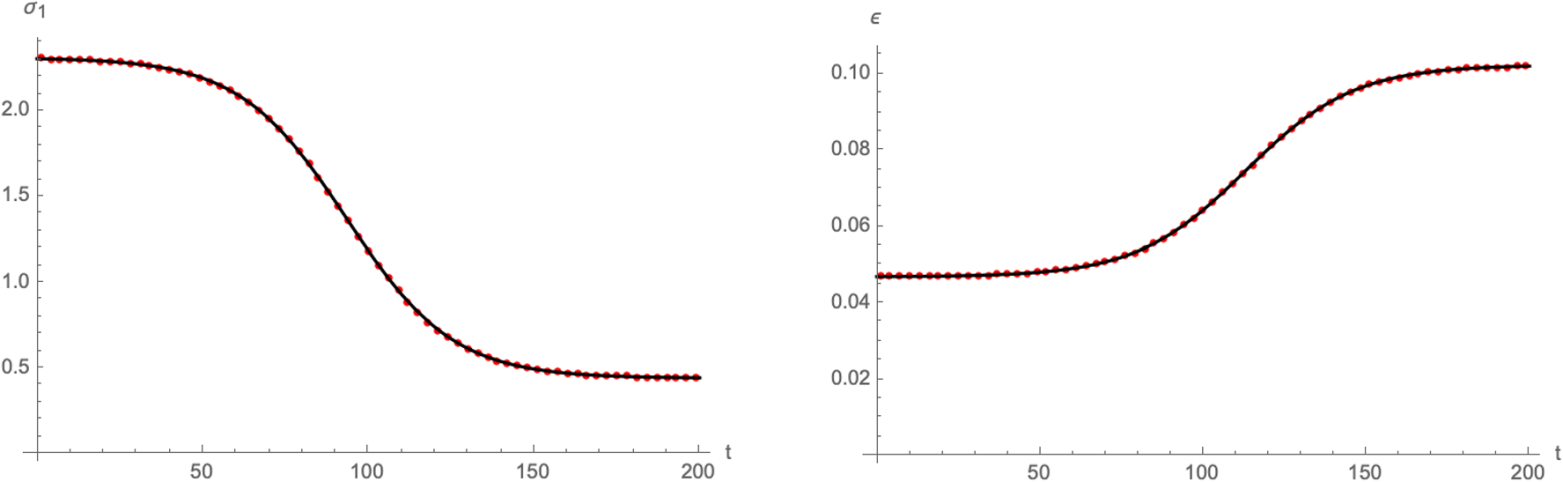
Time dependence of the infection rate $\sigma _1$ (left panel) and the removal rate $\varepsilon$ (right panel) needed to reproduce and $I_{\text {c}}(t)$ of the form (9) with the compartmental model (1) (red dots). Both plots use $A_0=0.025$, $\lambda _0=0.06$, $t_0=100$, $\tau =14$, $\rho =0$, $\zeta =0$, $\sigma _2/\sigma _1=1$ and $V_0=0$. The interpolating black lines correspond to approximations with logistic functions following (11) with notably $A_\sigma =1.87$, $\delta _\sigma =0.44$, $A_\varepsilon =0.055$ and $\delta _\varepsilon =0.047$.

As the eRG only describes the cumulative number of infected individuals $I_{\text {c}}$, in order to match the solutions to those of the SIIRV compartmental model introduced in the previous subsection, we need to make a further assumption on how the cumulative number of infected individuals is distributed among removed and infectious individuals. To this end we assume that

10 $$\begin{aligned} I(t)=\int _{t-\tau }^tdt\,\frac{dI_{\text {c}}(t')}{dt'}\,dt'=I_{\text {c}}(t)-I_{\text {c}}(t-\tau )\,, \end{aligned}$$

represents the total number of infectious individuals (either vaccinated or unvaccinated) at time *t*, where $$\tau$$ is the average amount of time an infectious individual remains infectious. We have verified that this assumption does not play any crucial role in the following analysis, and that it is compatible with the epidemiological data in Germany and Austria (see Supplementary Material for more details). Furthermore, in order to reproduce the cumulative number of infected (9) along with $$I(t)=N(I_1+I_2)(t)$$ in (10) with the SIIRV model (1) and (2), it is generally required that the infection and recovery rates of the compartmental model are functions of time^30^. We first validated the matching in a simple scenario, with fixed ratio $$\sigma _2/\sigma _1$$ and in absence of vaccinations (i.e. $$\rho =0$$ and $$V_0=0)$$. An typical example of time-dependent $$(\sigma _1,\varepsilon )$$ matching to the eRG solution is shown in Fig. 2. Functions of this form were previously found^30^ when matching the eRG approach to a time-dependent SIR model. More precisely, as is showcased by the black interpolating lines in Fig. 2, the time dependence of $$(\sigma _1(t),\varepsilon (t))$$ can be approximated by logistic functions of the form

11 $$\begin{aligned}&\sigma _1(t)=A_\sigma \left( 1-\frac{1}{1+e^{-\lambda _\sigma (t-t_\sigma )}}\right) +\delta _\sigma \,,&\varepsilon (t)=\frac{A_\varepsilon }{1+e^{-\lambda _\varepsilon (t-t_\varepsilon )}}+\delta _{\varepsilon }\,, \end{aligned}$$

where $\lambda _\sigma \sim \lambda _\varepsilon \sim \lambda _0$ and $t_\sigma \sim t_\varepsilon \sim t_0$ while $(A_\sigma ,\delta _\sigma ,A_\varepsilon ,\lambda _\varepsilon )$ show a more complicated dependence on $(A_0,\lambda _0,t_0)$.

#### Vaccinations

Next we consider a non-vanishing number of (fully) vaccinated individuals at the outbreak of the wave, $V_0\ne 0$, and a non-trivial vaccination rate $\rho$. We first study the impact of $V_0$ on the eRG model ($\rho = 0$) when it remains below the herd immunity threshold. We find convenient to match the solutions of the SIIRV model to a logistic function in the form

12 $$\begin{aligned} I_{\text {c}}(t)=\frac{A_0(1-\kappa V_0)}{1+e^{-\lambda _0(t-t_0)}}\,, \end{aligned}$$

where $\kappa$ is a numerical parameter close in value to $h^{\text {HIT}}$. As explained in the Supplementary material, this is still accomplished by a functional dependence of the form (11) for $(\sigma _1(t),\varepsilon (t))$ with a roughly linear dependence of $A_\sigma$ on $V_0$.

Finally we turn on the vaccination rate $\rho$. For the eRG framework, the dependence of $I_{\text {c}}$ on the vaccination rate has been discussed^52^, and it has been proposed to promote $\lambda _0$ and $A_0$ to dynamical functions of time that follow the additional first-order differential equations

13 $$\begin{aligned}&\frac{d\lambda _0}{dt}=-\rho \lambda _0(t=0)\,,&\text {and} \qquad&\frac{dA_0}{dt}=-\rho \,\left( A_0(t)-I_{\text {c}}(t)\right) ^w\,. \end{aligned}$$

in supplement to the $\beta$-function (8). This implies $\lambda _0(t)=\lambda _0(t=0)\left[ 1-t\,\rho \right]$, while the second equation in (13) for $w=1$ derives from the fact that, at any given time *t*, the reduction of the asymptotic cumulative number of infected individuals can only depend on remaining number of susceptible $A_0(t)-I_{\text {c}}(t)$^52^. As further explained in Section S4 of the Supplementary Material, here we find that a better match with the solutions of the SIIRV model can be obtained with $w\in [0,1)$.

#### Health pass models

Having established a correspondence between the eRG approach and the SIIRV model (1) and (2) with non-constant rates, we next assume that the time dependence of $(\sigma _1,\varepsilon )$ remains valid also after implementing either of the two HP  models (4) or (6) and the only modification is due to the (constant) parameter $p_{\text {VT}}$ and $p_{\text {V}}$ respectively. In this case, numerical solutions indicate that the cumulative number of infected individuals can still be well approximated by a logistic function (9) (see the left panel of Fig. 4), albeit with *p*-dependent parameters $(A_0,\lambda _0,t_0)$ as shown in the right panel of Fig. 4.

### Ethical approval

The methods employed in this work are in accordance with all the relevant guidelines and regulations.

## Results

### General results

**Figure 3:**
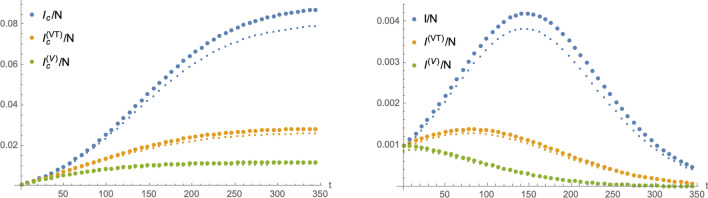
Numerical solutions of the SIIRV model with different variants of a HP: solutions of Eq. (1) are represented by blue points, those of Eq. (4) by orange points and those of (6) by green points. The left panel shows the cumulative number of infected individuals (large points stand for the total numbers, while small points represent only the unvaccinated individuals) and the right panel the infectious individuals as functions of time. Both plots use $\sigma _1=\sigma _2=1.6$, $\varepsilon =0.1$, $\zeta =0.15$, $\rho =0.0005$ and $V_0=0.3$.

**Figure 4:**
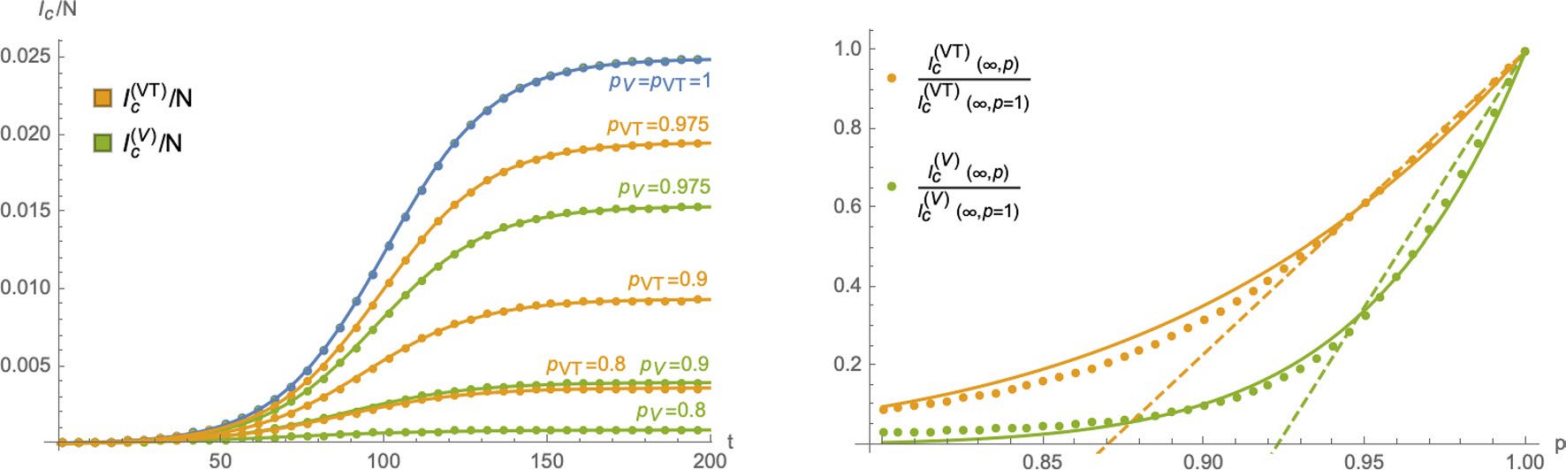
Left panel: cumulative number of infected in the two compartmental HP-models with time dependent parameters $(\sigma _1,\varepsilon )$ as a function of $p_{\text {VT}}$ and $p_{\text {V}}$: orange curves represent the model (4) and green curves the model (6). The blue curve (with $p_{\text {VT}}=p_{\text {V}}=1$) is identical in both models (and corresponds to the case of no HP  in (1)). Right panel: comparison of the asymptotic cumulative number of infected individuals as a function of *p* (and normalised to $p=1$) for (4) and (6). The dots represent the numerical solutions, while the dashed lines stand for the leading (linear) approximation at $p=1$ and the solid lines for interpolations with exponential functions of the form (14). The plot uses $\sigma _1=\sigma _2=1.6$, $\varepsilon =0.1$, $\zeta =0.15$, $\rho =0.0005$ and $V_0=0.3$ and leads to the interpolation parameters $\theta ^{(\text {VT})}=9.326$ and $\theta ^{(\text {V})}=20.364$.

Numerical solutions of the SIIRV model (1) and (2) as well as the two HP-modifications (4) and (6) are shown in Fig. 3. The right panel of this figure demonstrates the potential of a HP  to ‘flatten the curve’, i.e. to reduce the local maximum of the number of infectious individuals as a function of time, or even completely eliminate it. To further study this point, the left panel of Fig. 4 shows the cumulative number of infected individuals for the two compartmental HP-models (4) and (6). The right panel of this figure shows a comparison of their asymptotics (i.e. at the end of the wave) as a function of *p* (and normalised to $p=1$). For a certain range of $p<1$, the numerical solutions can be interpolated by exponential functions of the form

**Figure 5:**
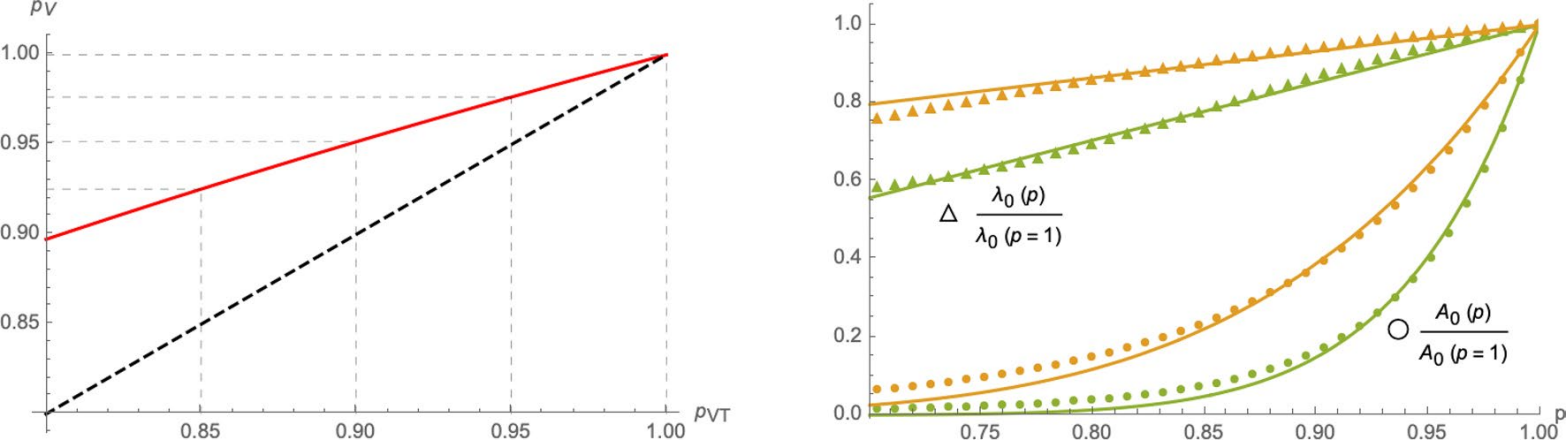
Left panel: using the same numerical parameters as in Fig. 4, the red curve represents the relation between the *p*-parameters of (4) and (6) that lead to the same asymptotic cumulative number of infected individuals. The dashed black line represents for comparison the relation $p_{\text {V}}=p_{\text {VT}}$. Right panel: *p*-dependence of the parameters $(A_0,\lambda _0)$ relative to the case $p=1$ in the eRG approach equivalent to the SIIRV model with time dependent rates: circles represent numerical values of $\frac{A_0(p)}{A_0(p=1)}$ while triangles represent numerical values of $\frac{\lambda _0(p)}{\lambda _0(p=1)}$, with orange symbols computed using the model (4) and green symbols correspond to the model (4). The solid lines represent interpolations of the numerical solutions with an exponential function of the form (16) for $\frac{A_0(p)}{A_0(p=1)}$ and a linear function for $\frac{\lambda _0(p)}{\lambda _0(p=1)}$. The plots use $A_0=0.025$, $\lambda _0=0.06$ (at $t=0$), $t_0=100$, $V_0=0.3$ and $\zeta =0.15$.

14 $$\begin{aligned}&I_{\text {c}}^{(\text {VT},\text {T})}(\infty ,p)\sim I_{\text {c}}^{(\text {VT},\text {T})}(\infty ,p=1)\,\text {exp}\left( \theta ^{(\text {VT},\text {T})}\,\frac{p-1}{p}\right) \,,&\text {with}\qquad&\theta ^{(\text {VT},\text {T})}\in {\mathbb {R}}_+\,, \end{aligned}$$

with the two models mainly differing by the constant fitting parameters $\theta ^{(\text {VT})}$ and $\theta ^{(V)}$. In fact, approximations of this type are already viable within the framework of a SIR model^14^ without any vaccination dynamics. More details, including a comparison of (14) with the first and second order of a Taylor series expansion around $p=1$ can be found in the Supplementary Material. Assuming all remaining parameters to be the same (notably the recovery rate $\varepsilon$), the same efficacy of the HP-models (4) and (6), $p_{\text {VT}}$ and $p_{\text {V}}$ respectively, lead to different asymptotic cumulative numbers of infected individuals. We can turn this relation around by determining which values of $p_{\text {VT}}$ and $p_{\text {V}}$ (for all other parameters being held fixed), lead to the same number of infected individuals at the end of the epidemic wave. The red line in the left panel of Fig. 5 shows the relation between $p_{\text {V}}$ and $p_{\text {VT}}$ that needs to be satisfied in order to obtain the same asymptotic behaviour: for the parameters chosen, $1-p_{\text {VT}}$ in a HP  that accepts both test and vaccination certificates needs to be roughly a factor 2 larger than $1-p_{\text {V}}$ in a HP , which only allows vaccinated individuals full access to public life. The relation in the left panel of Fig. 5 can be studied using the approximation (14), which implies equivalent asymptotic numbers of infected individuals for

15 $$\begin{aligned} p_{\text {V}}&=\frac{p_{\text {V}}\,\theta ^{(\text {V})}}{p_{\text {VT}}(\theta ^{(\text {V})}-\theta ^{(\text {VT})})+\theta ^{(\text {VT})}}=1+\frac{\theta ^{\text {(VT)}}}{\theta ^{\text {V}}}\,(p_{\text {VT}}-1)+\frac{\theta ^{(\text {VT})}(\theta ^{(\text {VT})}-\theta ^{(\text {V})})}{(\theta ^{(\text {V})})^2}\,(p_{\text {VT}}-1)^2+{\mathscr {O}}((p_{\text {VT}}-1)^3)\,. \end{aligned}$$

The linear approximation around $p_{\text {VT}}=1$ with the coefficient $\frac{\theta ^{\text {(VT)}}}{\theta ^{\text {V}}}\sim 0.458$ indeed very well agrees with the left panel of Fig. 5. We stress, however, that this comparison assumes that all remaining parameters of the system remain the same for both models. In particular, we assumed the same removal rate $\varepsilon$ in both cases, which (among other things) depends on the efficiency of the contact-tracing, i.e. identifying and quarantining infected individuals and therefore also crucially depends on the number of tests that are being performed per time unit. Since a V-HP-model offers less incentive for individuals to get tested (unless they present clear symptoms), they likely also entail a lower test rate, leading ultimately to a smaller value of $\varepsilon$. A numerical equivalence taking into account a possible change in $\varepsilon$ is discussed in Section S5 of the Supplementary Material.

From the perspective of the eRG, which is equivalent to the SIIRV models (4) and (6) with time dependent infection and removal rates of the form (11), relation (14) implies that a HP can be implemented by allowing for a *p*-dependence of $A_0$ and $\lambda _0$ in the $\beta$-function (8), concretely

16 $$\begin{aligned}&A_0(p)\sim A_0(p=1)\,\text {exp}\left( \theta \,\frac{p-1}{p}\right) \,,&\qquad \text {with}\qquad&\theta \in {\mathbb {R}}_+. \end{aligned}$$

The constant $\theta$ implicitly depends on the remaining parameters of the problem (notably the vaccination dynamics and $A_0(p=1)$). The *p* dependence of $\lambda _0$ for small $1-p$ can be approximated to be linear, as is shown in the right panel of Fig. 5.

### Examples

We next apply the theoretical results developed above to the epidemiological situation of Germany and Austria in the late summer/early fall of 2021. Further examples of France, Italy and Denmark are discussed in Section S6 of the Supplementary Material.

#### Germany

The epidemiological situation in Germany since the beginning of the COVID-19 pandemic and the summer of 2021 is shown in Section S6.2 of the Supplementary Material. The available data for an impending wave from 07/July/2021 to 17/August/2021 can be fitted with a logistic function of the form

17 $$\begin{aligned} I_{\text {c}}^{\text {wave}}(t)=I_{\text {c},0}+\frac{A_0}{1+e^{-\lambda _0(t-t_0)}}\,, \end{aligned}$$

which however shows a large uncertainty when extrapolated until the middle of September. We therefore consider as two extremal cases logistic functions parametrised by (for more information see the Supplementary Material)

18 $$\begin{aligned}&A_0^+=2.5\cdot 10^6\,,&\lambda _0^+=0.072\,,&\quad t_0^+=594.5\,,&\delta _0^+=3.74\cdot 10^6\,,\nonumber \\&A_0^-=174533\,,&\lambda _0^-=0.094\,,&\quad t_0^-=548.3\,,&\delta _0^-=3.74\cdot 10^6\,. \end{aligned}$$

**Figure 6:**
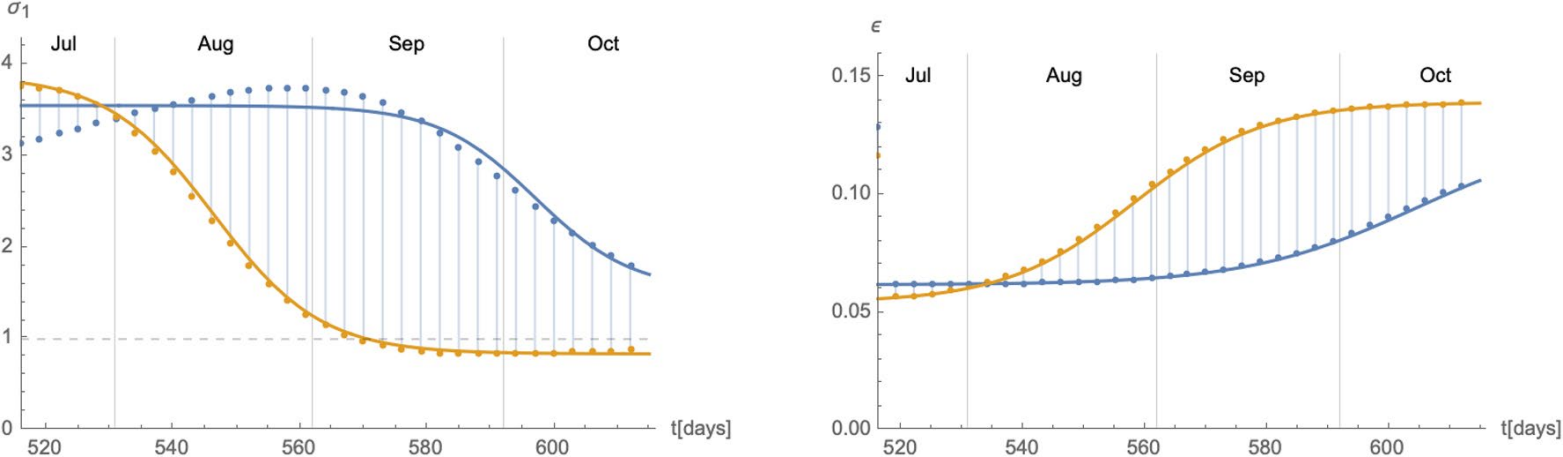
Time dependent parameters $\sigma _1$ (left panel) and $\varepsilon$ (right panel) for the extremal cases of wave 4. The blue and orange colours are correlated with the curves in the right panel of Fig. F10 in the Supplementary Material.

Based on these values, we can develop a time-dependent SIIRV model, with time dependent parameters $(\sigma _1,\varepsilon )$, which are shown in Fig. 6. These curves follow the general form of Eq. (11) with the parameters

**Table:** 

	$A_{\sigma ,\varepsilon }^+$	$A_{\sigma ,\varepsilon }^-$	$\lambda _{\sigma ,\varepsilon }^+$	$\lambda _{\sigma ,\varepsilon }^-$	$t_{\sigma ,\varepsilon }^+$	$t_{\sigma ,\varepsilon }^-$	$\delta _{\sigma ,\varepsilon }^+$	$\delta _{\sigma ,\varepsilon }^-$
$\sigma _1$	2.024	3.047	0.131	0.119	81.1	30.4	1.529	0.838
$\varepsilon$	0.065	0.085	0.072	0.094	88.5	42.3	0.062	0.054

**Figure 7:**
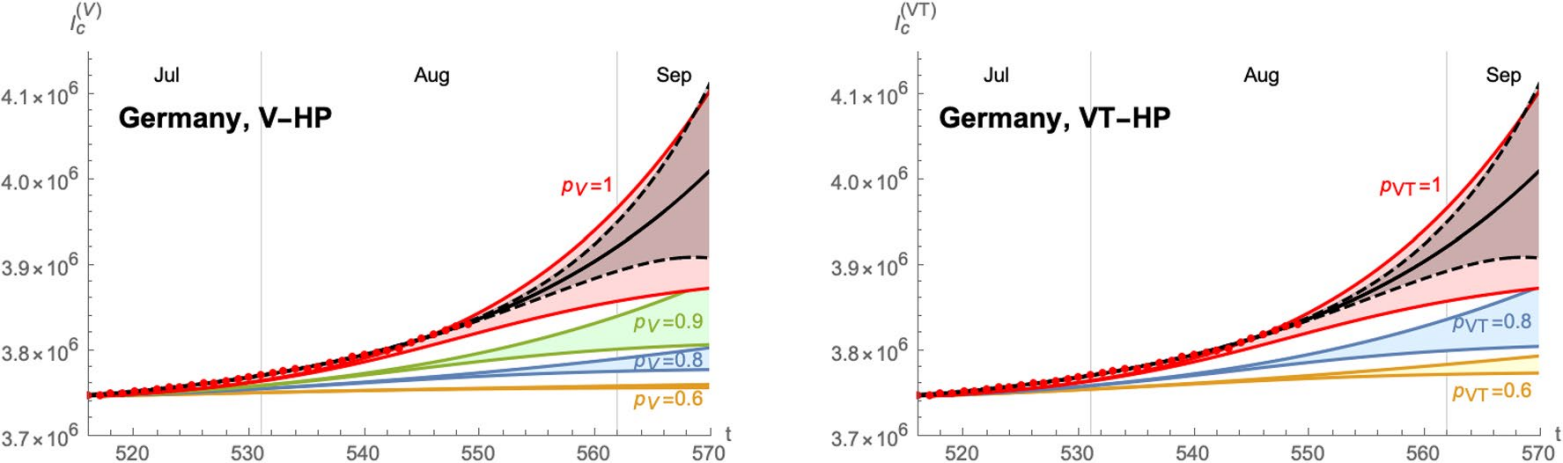
Time evolution of the cumulative number of infected individuals for different values of the efficacy of a V-HP  (right panel) or a VT-HP (right panel). We assume that the model has been introduced on 07/07/2021. Both cases use $\zeta =0.15$ (based on an average of the efficacy of each vaccine weighted by the distribution among the population), $\rho =0.008$ and $\sigma _2/\sigma _1=1$.

**Figure 8:**
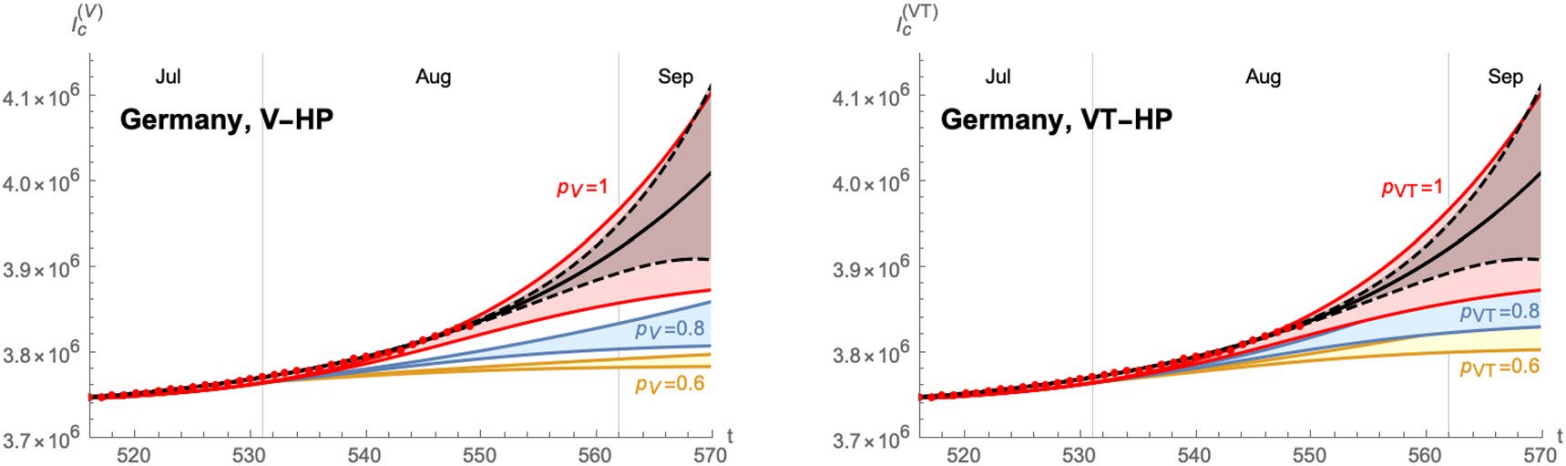
Time evolution of the cumulative number of infected individuals for different values of the efficacy of a V-HP  (right panel) or a VT-HP (right panel). We assume that the model has been introduced on 01/08/2021. Both cases use $\zeta =0.15$ (based on an average of the efficacy of each vaccine weighted by the distribution among the population), $\rho =0.008$ and $\sigma _2/\sigma _1=1$.

**Figure 9:**
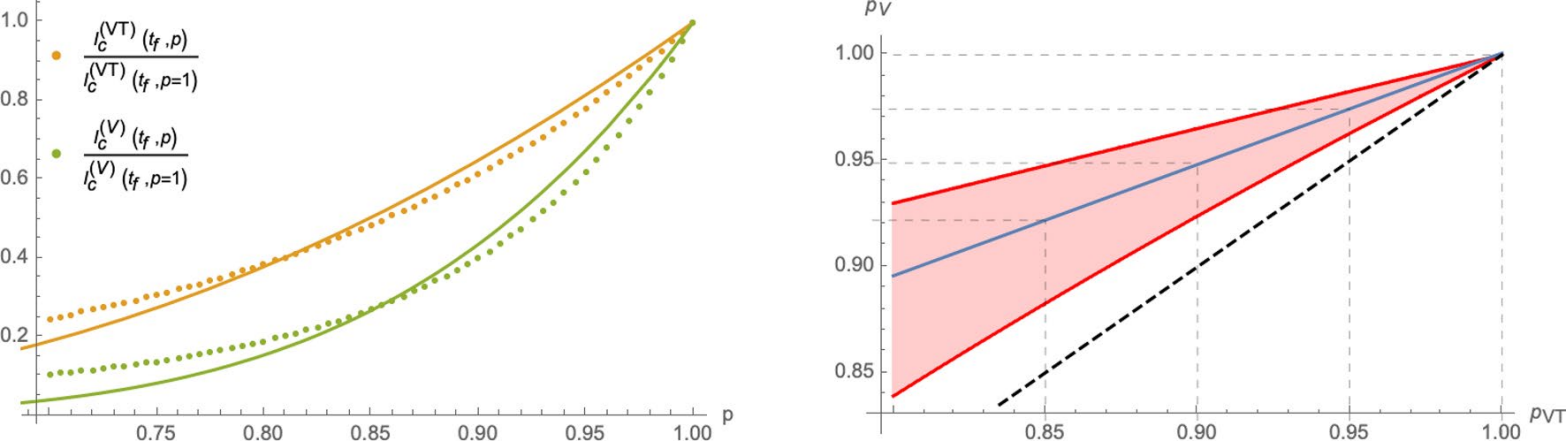
Comparison of the V-HP  model (6) and the VT-HP  model (4): the left panel shows the cumulative number of infected individuals at $t_f=15/09/2021$ as a function of *p* (normalised to the value of $p=1$). The orange curve corresponds to the model (6) and the green curve to the one in (4). The right panel shows the equivalence for the parameters $p_{\text {VT}}$ and $p_{\text {V}}$ of these two models, taking into account the incertitude inherent in the approximations: the red band indicates equivalent values of these parameters that lead to the same value of $I_{\text {c}}(t_f)$ with the blue line corresponding to equivalence obtained comparing equivalent extrapolations of the data in each case.

Finally, implementing the Green pass model based on these extremal cases is shown in Fig. 7. Starting from the band of cumulative number of infected individuals (for $p=1$), we obtain a similar band for each $p<1$. The plots for a VT-HP  and V-HP  respectively are shown for $\sigma _2/\sigma _1=1$ in Fig. 7, assuming that the HP  has been introduced on 07/July/2021. Figure 8 shows the same analysis assuming that the HP  had been introduced on 01/August/2021.The plots in Fig. 7 suggest that a new wave in Germany could be stopped by reducing the contacts among non-vaccinated individuals by roughly 20–40%. Finally, we have compared the efficacy of the VT-HP  and V-HP  in the case of Germany in Fig. 9: the left panel shows the (normalised) cumulative number of infected individuals at $t_f=15/September/2021$, along with an approximation of the form (16). Notice, rather than the asymptotic number of infected individuals at the end of the wave, we have chosen a date roughly a months after the last available data for the comparison. The right panel shows which values of $p_{\text {V}}$ and $p_{\text {VT}}$ lead to the same cumulative number of infected individuals at $t_f$: the red band corresponds to the uncertainty related to the two extremal cases we have developed to extrapolate the data. In fact, the extrema of this band arise when comparing the most optimistic extrapolation for the V-HP  with the worst case approximation of VT-HP  (and vice versa). The blue line corresponds to a comparison of equivalent extrapolations and suggests roughly

19 $$\begin{aligned} 2(1-p_{\text {V}})\sim 1-p_{\text {VT}}\,. \end{aligned}$$

This means, assuming that all other parameters remain roughly the same, the reduction in the contacts in the VT-HP  needs to be roughly twice as large as in the V-HP-model to achieve the same cumulative number of infected.

#### Austria

**Figure 10:**
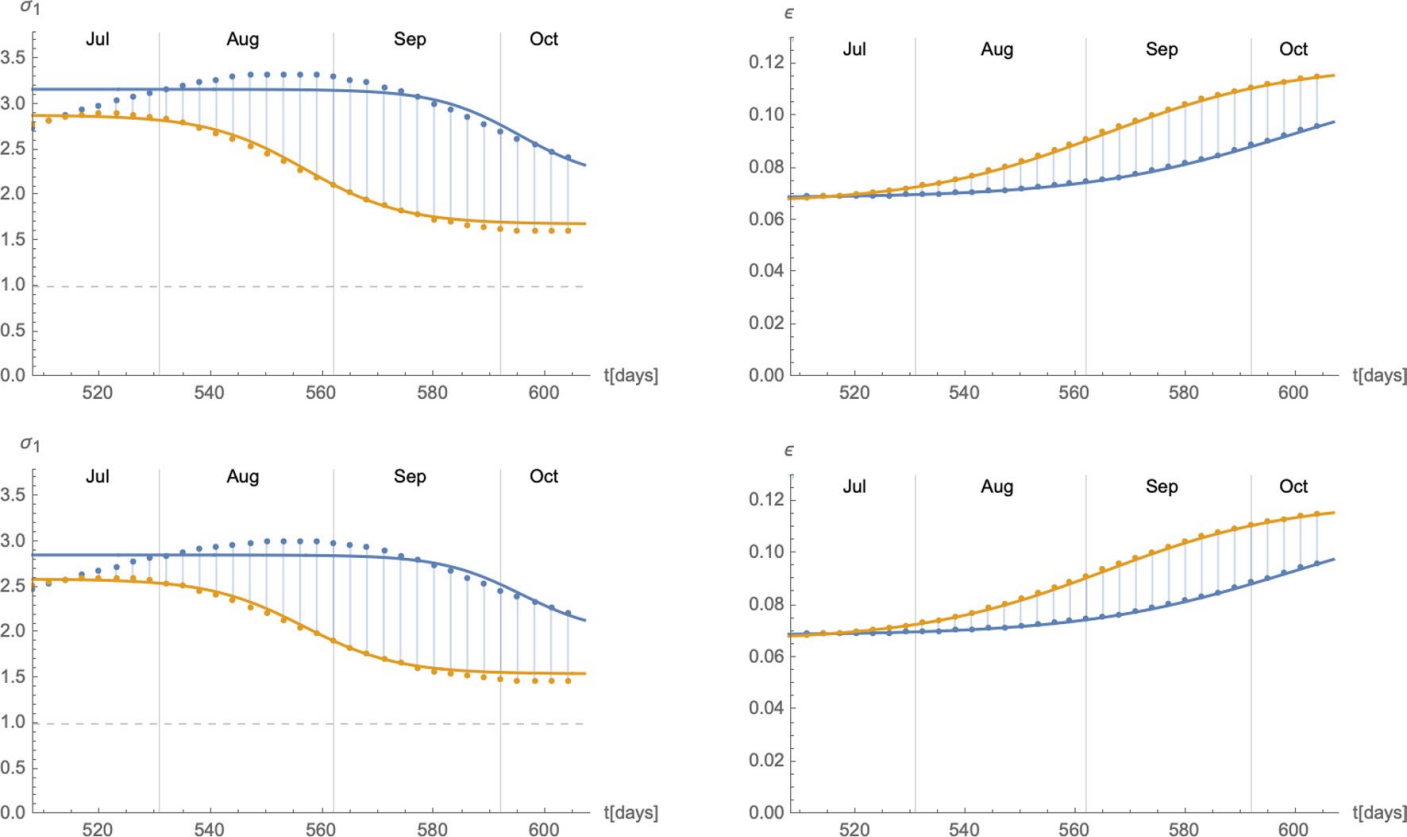
Time dependent parameters $\sigma _1$ (left panel) and $\varepsilon$ (right panel) for the extremal cases of wave 4. The blue and orange colours are correlated with the curves in the right panel of Figure F10. The panels of the top row use $p=0.8$, while the panels in the bottom row use $p=0.9$ in order to fit the data.

**Figure 11:**
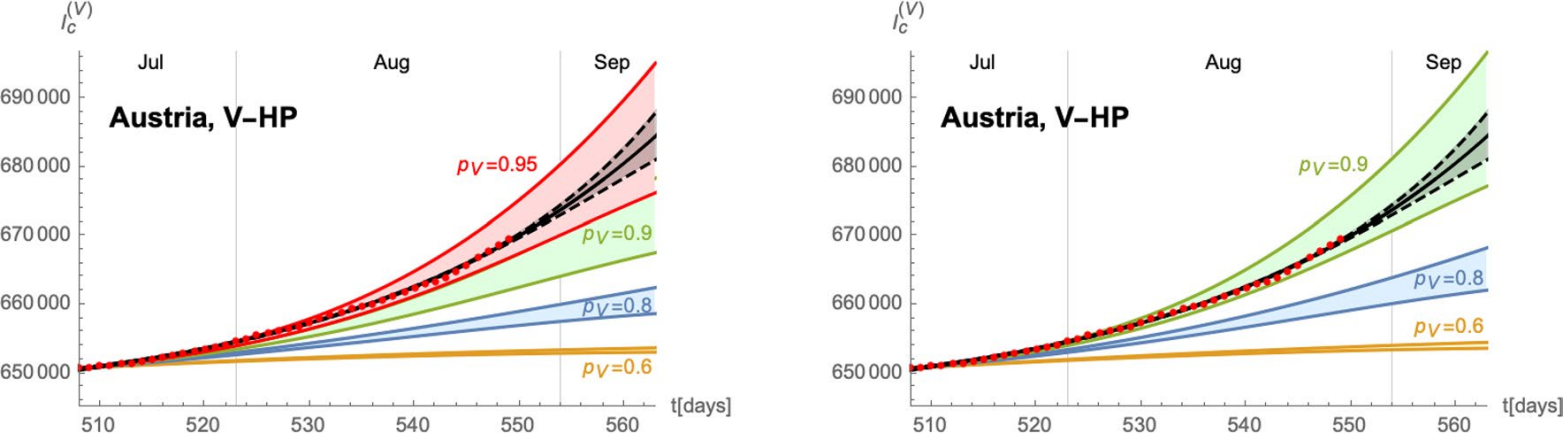
Predicted time evolution of the cumulative number of infected individuals for different values of the efficacy $p_{\text {V}}$ of the V-HP model. The left panel assumes $p_{\text {VT}}=0.9$ for the VT-HP  currently in place in Austria and the right panel $p_{\text {VT}}=0.8$. Both plots assume furthermore $\sigma _2/\sigma _1=1$ and $\rho =0.0085$.

The epidemiological situation in Austria since the beginning of the COVID-19 pandemic and the summer of 2021 is shown in Section S6.3 of the Supplementary Material. As in Germany, the available data from 01/July/2021 to 17/August/2021 show a great uncertainty when extrapolated until middle of September. We therefore again fit two extremal logistic functions of the form (17), with the exact numerical data given in Section S6.3 of the Supplementary Material. These data suggest the onset of a new wave, just as in the case of Germany. However, unlike Germany, a VT-HP  (3-G-rule: ‘geimpft, getestet, genesen’) was enforced on 01/July/2021, with earlier measures dating as far back as 19/May/2021 (the rule was slightly modified on 22/07 and 15/08 specifying stricter rules to discotheques and nightclubs and imposing restrictions to only partially vaccinated individuals respectively, see the Supplementary Material for further details) allowing individuals full access to the public life only with a certificate of either being (fully) vaccinated, having recovered from a previous infection or having tested negative for SARS-Cov-2 . Therefore, in order to derive time-dependent parameters $(\sigma _1(t),\varepsilon (t))$ for the above mentioned extremal cases, we need to take the presence of the V-HP  into account. Since the exact efficacy of the HP  are difficult to quantify, we have used $p=0.8$ and $p=0.9$ as reference values to fit the data. The corresponding time-dependent functions $(\sigma _1(t),\varepsilon (t))$ for wave 4 are shown in Fig. 10. As is evident, the main difference lies in the function $\sigma _1$, while the curve for $\varepsilon$ is relatively unchanged. Indeed, as remarked before, mathematically the parameter $p_{\text {VT}}$ can be absorbed in the $\gamma _1$. Since we assumed for the latter anyway a certain range, the main effect of $p_{\text {VT}}$ can also be absorbed in the quotient $\sigma _2/\sigma _1$, i.e. the reduction in the rate at which vaccinated infectious individuals infect others. We next apply these time-dependent $(\sigma _1,\varepsilon )$ parameters to the stronger V-HP  model (6). The results are shown in Fig. 11. The results again support an equivalence of the type (19) between the parameters $p_{\text {V}}$ of the V-HP  and $p_{\text {VT}}$ of the VT-HP model.

## Conclusions

In this paper we have analysed the impact of Health Passes on the epidemiological dynamics of infectious diseases. These HPs correspond to measures that restrict the access of individuals with a higher risk of being infectious to public life. Concretely, we have distinguished two different classes that grant access to individuals with a vaccination certificate or a recent negative test (VT-HP) and only to vaccinated individuals (V-HP).

We have first discussed these HPs in the context of a simple compartmental SIIRV model (1) and have generalised them in the context of the eRG framework, which is better suited for describing the dynamics over a longer period of time, in particular an entire epidemiological wave. Analysing in particular the dependence of the asymptotic cumulative number of infected individuals (which is a crucial parameter in the description of the eRG), we have found the approximative exponential dependence (16) on the parameter describing the efficacy of the HP . Furthermore, comparing the efficacy of a VT-HP-model to a V-HP  model reduces to comparing the corresponding $\theta$-parameters appearing in this approximation.

We have furthermore validated our models by discussing the diffusion of COVID-19 in several European countries. We have analysed in detail Germany (who, to this date, has not implemented any HP) and Austria (who currently has implemented a VT-HP  and considers the partial introduction of a V-HP) and have presented a briefer analysis for France, Denmark and Italy. In all cases we have established that a V-HP  is much more efficient in reducing the number of infected. Our model in fact allows for a quantitative comparison, leading to the relation (19): if all remaining parameters remain the same, the efficacy of a VT-HP  needs to roughly be twice as high to produce the same reduction of infections as a V-HP . Furthermore in most cases, an efficiency of a V-HP  of roughly 20-40% is strong enough to completely suppress a potential fourth wave.

We have undertaken preliminary studies that also include a potential reduction in the number of tests (related to a reduction in the removal rate due to a reduced capacity of identifying and isolating infected individuals). It would be important to further extend these studies, in particular to establish a quantitative relation between these two rates.

## Supplementary Information

Supplementary Information.

## Data Availability

The epidemiological data are extracted from the open-source repository on Worldometer. Data about the vaccination rates and progression have been downloaded from the Robert Koch Institute for Germany^55^, and from the Austrian Ministry webpage for Austria^56^.
