# Supplementary material for: Effective mathematical modelling of health passes during a pandemic
Source: Sci Rep. 2022 Apr 28;12:6989. doi: 10.1038/s41598-022-10663-5 (PMC9049016; doi:10.1038/s41598-022-10663-5)
Supplement: Supplementary file 1 — Supplementary Information. [file 41598_2022_10663_MOESM1_ESM.pdf]

Effective Mathematical Modelling of Health Passes during a Pandemic

Stefan Hohenegger[1,2], **Giacomo Cacciapaglia**[1,2,*], **and Francesco Sannino**[3,4,5]

[1]Institut de Physique des 2 Infinis (IP2I) de Lyon, CNRS/IN2P3, UMR5822, 69622 Villeurbanne, France
[2]Université de Lyon, Université Claude Bernard Lyon 1, 69001 Lyon, France
[3]Scuola Superiore Meridionale, Largo S. Marcellino, 10, 80138 Napoli NA, Italy
[4]Dipartimento di Fisica, E. Pancini, Univ. di Napoli, Federico II and INFN sezione di Napoli, Complesso Universitario di Monte S. Angelo Edificio 6, via Cintia, 80126 Napoli, Italy
[5]CP[3]-Origins and D-IAS, Univ. of Southern Denmark, Campusvej 55, DK-5230 Odense, Denmark
[*]g.cacciapaglia@ipnl.in2p3.fr

ABSTRACT

We study the impact on the epidemiological dynamics of a class of restrictive measures that are aimed at reducing the number of contacts of individuals who have a higher risk of being infected with a transmittable disease. Such measures are currently either implemented or at least discussed in numerous countries worldwide to ward off a potential new wave of COVID-19. They come in the form of Health Passes (HP), which grant full access to public life only to individuals with a certificate that proves that they have either been fully vaccinated, have recovered from a previous infection or have recently tested negative to SARS-Cov-2. We develop both a compartmental model as well as an epidemic Renormalisation Group approach, which is capable of describing the dynamics over a longer period of time, notably an entire epidemiological wave. Introducing different versions of HPs in this model, we are capable of providing quantitative estimates on the effectiveness of the underlying measures as a function of the fraction of the population that is vaccinated and the vaccination rate. We apply our models to the latest COVID-19 wave in several European countries, notably Germany and Austria, which validate our theoretical findings.

Supplementary Material

S1 Analytic Results for the SIIRV Compartmental Model

S1.1 SIIRV Model for Vanishing Vaccination Rate

While the differential equations (1) are in general difficult to solve analytically, there are a few results we can derive in the case of vanishing vaccination rate: we start with the system (1) for $\rho = 0$ and divide the differential equation for $\frac{dS}{dt}$ by the differential equation for $\frac{dV}{dt}$ to obtain $\frac{dS}{dV} = \frac{S}{\zeta V}$, which has the following solution compatible with the initial conditions

$$\frac{V(t)}{V_0} = \left(\frac{S(t)}{S_0}\right)^{\zeta}, \qquad \forall t \in \mathbb{R}. \tag{E1}$$

Inserting this relation into the differential equations for $\frac{dI_1}{dt}$ and $\frac{dI_2}{dt}$ in (1) we obtain

$$\frac{dI_1}{dt} = S\,[\gamma_1 I_1 + \gamma_2 I_2] - \varepsilon I_1\,, \qquad \text{and} \qquad \frac{dI_2}{dt} = \zeta\, V_0 \left(\frac{S}{S_0}\right)^{\zeta} [\gamma_1 I_1 + \gamma_2 I_2] - \varepsilon I_2\,. \tag{E2}$$

The combination of these two equations implies

$$\frac{d(\gamma_1 I_1 + \gamma_2 I_2)}{dt} = \left(\gamma_1 S + \gamma_2 \zeta\, V_0 \left(\frac{S}{S_0}\right)^{\zeta} - \varepsilon\right)(\gamma_1 I_1 + \gamma_2 I_2)\,, \tag{E3}$$

which has the following solution compatible with the initial conditions

$$(\gamma_1 I_1 + \gamma_2 I_2)(t) = (\gamma_1 I_{1,0} + \gamma_2 I_{2,0}) \exp\left[\int_0^t dt' \left(\gamma_1 S(t') + \gamma_2\, \zeta\, V_0 \left(\frac{S(t')}{S_0}\right)^{\zeta} - \varepsilon\right)\right]. \tag{E4}$$

Inserting this result into the first equation of (1) gives an integro-differential equation for S

$$\frac{dS}{dt}(t) = -S(t)\,(\gamma_1 I_{1,0} + \gamma_2 I_{2,0})\exp\left[\int_0^t dt'\left(\gamma_1\,S(t') + \gamma_2\,\zeta\,V_0\left(\frac{S(t')}{S_0}\right)^{\zeta} - \varepsilon\right)\right] \tag{E5}$$

which leads to the following (non-linear) second order differential equation

$$\frac{d}{dt}\left(\ln\left(\frac{d\ln S}{dt}\right)\right) = \gamma_1\,S + \gamma_2\,\zeta\,V_0\left(\frac{S}{S_0}\right)^{\zeta} - \varepsilon\,. \tag{E6}$$

The latter yields a solution for S, which, when injected together with (E4) into the first equation of (E2) yields an ordinary differential equation for I_1, thus decoupling the initial system (1).

In order to get information about the asymptotic behaviour of the number of susceptible, we return to eq. (E3) and divide by the first equation of (1)

$$\frac{d(\gamma_1\,I_1 + \gamma_2 I_2)}{dS} = -\frac{1}{S}\left(\gamma_1\,S + \gamma_2\zeta\,V_0\left(\frac{S}{S_0}\right)^{\zeta} - \varepsilon\right). \tag{E7}$$

Instead of a differential equation for the combination $\gamma_1\,I_1 + \gamma_2\,I_2$ as a function of time, we have an equation as a function of S, which has the following solution that is compatible with the initial conditions

$$(\gamma_1\,I_1 + \gamma_2 I_2) = \gamma_1\,I_{1,0} + \gamma_2\,I_{2,0} - \gamma_1\,(S - S_0) - \gamma_2\,(V - V_0) + \varepsilon\,\ln\left(\frac{S}{S_0}\right). \tag{E8}$$

For asymptotic times, we have $\lim_{t\to\infty} I_1(t) = 0 = \lim_{t\to\infty} I_2(t)$ such that we obtain the following equation for $S_\infty = \lim_{t\to\infty} S(t)$

$$\gamma_1\,I_{1,0} + \gamma_2\,I_{2,0} = \gamma_1\,(S_\infty - S_0) + \gamma_2\,V_0\left[\left(\frac{S_\infty}{S_0}\right)^{\zeta} - 1\right] - \varepsilon\,\ln\left(\frac{S_\infty}{S_0}\right), \tag{E9}$$

which can only be solved numerically for S_∞.

S1.2 Herd Immunity

An important parameter in the compartmental SIIRV model (1) (with initial conditions (2)) is the number of vaccinated individuals at the outbreak of the disease, V_0. Indeed, the asymptotic behaviour of the dynamics crucially depends on it and, for fixed $\gamma_{1,2}$, ε, ρ and ζ, the infinite-time limit $I_c(\infty)$ shows a critical behaviour with respect to this parameter: as shown in the numerical plots in Figure F1, the asymptotic cumulative number of infected individuals shows a linear decrease as a function of V_0 up to a certain (critical) value, above which the number of infected during the entire outbreaks remains relatively small (compared to the total size of the population).

The phenomenon that the spread of the disease is severely hampered if a certain critical percentage of the population has been immunised is called *herd immunity*. In the current model, we expect that this threshold depends on the various parameters. However, since the effect is most pronounced for vanishing vaccination rate ($\rho = 0$), we can derive a bound (called the herd immunity threshold) in this limit, using the analysis of the previous subsection for $\rho = 0$: To this end, we first derive the condition for the (relative) number of new infected to reach a local extremum. Indeed, the former can be defined as the time derivative of $I_c(t)$

$$I^{\text{new}}(t) = \frac{1}{N}\frac{dI_c}{dt}(t) = \frac{d}{dt}(1 - S - V)\,. \tag{E10}$$

A necessary condition for a local extremum of the (relative) number of new cases is therefore

$$0 = \frac{d^2}{dt^2}(S+V) = \frac{d^2S}{dt^2} + V_0\,\frac{d^2}{dt^2}\left(\frac{S}{S_0}\right)^{\zeta} = \left(1 + \zeta\frac{V}{S}\right)\frac{d^2S}{dt^2} + \zeta(\zeta-1)\frac{V}{S^2}\left(\frac{dS}{dt}\right)^2. \tag{E11}$$

where we have used (E1). We next use the differential equation (E6) to eliminate $\frac{d^2S}{dt^2}$

$$0 = \frac{1}{S}\left(\frac{dS}{dt}\right)\left[S\left(\gamma_1\,S + \gamma_2\,\zeta\,V - \varepsilon + \frac{1}{S}\frac{dS}{dt}\right) + \zeta V\left(\gamma_1\,S + \gamma_2\,\zeta\,V - \varepsilon + \frac{\zeta}{S}\frac{dS}{dt}\right)\right]. \tag{E12}$$

Since we are looking for a local extremum for $t < \infty$, we may assume $S(t) > 0$ and $V(t) > 0$. Therefore, the previous condition has two solutions

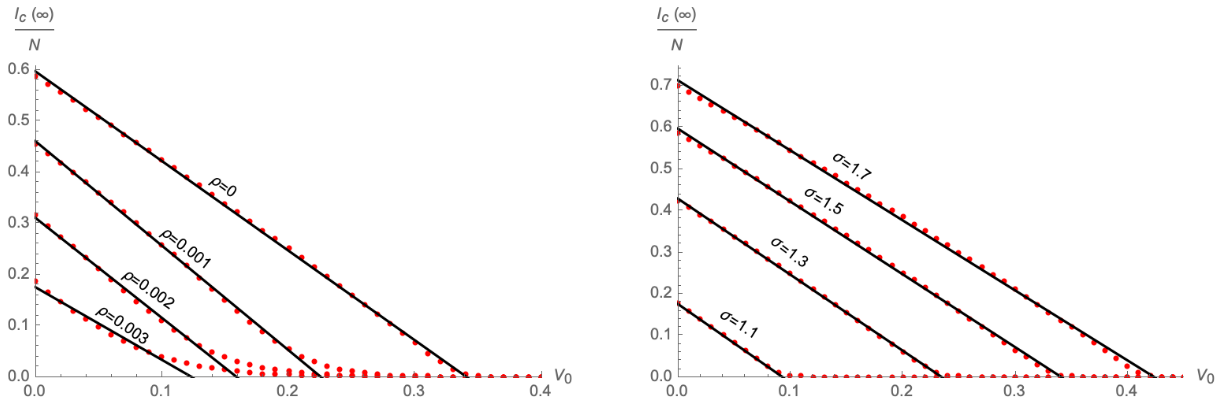

Figure F1. Left panel: Asymptotic cumulative number of infected individuals as a function of V_0 for different values of the vaccination rate ρ. The plot furthermore uses $\sigma_1 = \sigma_2 = 1.5$, $\varepsilon = 0.2$ and $\zeta = 0$. Right panel: Asymptotic cumulative number of infected individuals as a function of V_0 for different values of σ_1. The plot furthermore uses $\sigma_1 = \sigma_2 = \sigma$, $\varepsilon = 0.2$, $\rho = 0$ and $\zeta = 0$

- $\frac{dS}{dt} = 0$: with (E1) this condition also implies $\frac{dV}{dt} = 0$ and thus with (1)

$$S(t) = 0 = V(t)\,, \qquad \text{or} \qquad I_1(t) = 0 = I_2(t)\,, \tag{E13}$$

(since $I_i \geq 0$ for $i = 1,2$). The former relation corresponds to the case where the entire population has been infected, while the latter case corresponds to the eradication of the disease. Both cases constitute the end of the epidemic and are thus not local extrema

- $0 = S\left(\gamma_1 S + \gamma_2 \zeta V - \varepsilon + \frac{1}{S}\frac{dS}{dt}\right) + \zeta V \left(\gamma_1 S + \gamma_2 \zeta V - \varepsilon + \frac{\zeta}{S}\frac{dS}{dt}\right)$
Since we asssume $S > 0$ and $V > 0$, this relation can only hold if exactly one of the terms

$$\gamma_1 S + \gamma_2 \zeta V - \varepsilon + \frac{1}{S}\frac{dS}{dt}\,, \qquad \text{and} \qquad \gamma_1 S + \gamma_2 \zeta V - \varepsilon + \frac{\zeta}{S}\frac{dS}{dt}\,, \tag{E14}$$

is negative (and the other positive). However, since $I_{1,2} > 0$ and therefore (1) implies that S is a monotonically decreasing function, *i.e.* $\frac{dS}{dt} < 0$, this is only possible if $\gamma_1 S + \gamma_2 \zeta V - \varepsilon > 0$ and thus leads to the following necessary condition

$$\gamma_1 S_0 + \gamma_2 \zeta V_0 > \varepsilon\,. \tag{E15}$$

Notice, for $\zeta = 0$, we require $S(\gamma_1 S - \varepsilon) = 0$ leading to the necessary condition $\gamma_1 S_0 \geq \varepsilon$ for a local extremum, which is compatible with (E15).

The relation (E15) can also be formulated in terms of $\sigma_1 = \frac{\gamma_1}{\varepsilon_1}$ and $\sigma_2 = \frac{\gamma_2}{\varepsilon_2}$, namely $\sigma_1 S_0 + \zeta \sigma_2 V_0 > 1$. Notice, for $\zeta = 0$ (in which case V is decoupled from the time evolution), this condition is compatible with the usual threshold condition $\sigma_1 S_0 > 1$ of the SIR model.

In the SIIRV model (1), we define the herd immunity threshold h^{HIT} as the minimal fraction of the population that needs to be vaccinated beforehand, such that during the outbreak of the disease the number of new infections never reaches an extremum, but remains a monotonically decreasing function (For the outbreak, we shall consider the limit $I_{1,0} \to 0$ and $I_{2,0} \to 0$). To this end, we first define the vaccinated fraction of the population at the outbreak of the disease as $h = \frac{V_0}{S_0 + V_0}$. We furthermore consider initial conditions corresponding to the limit $I_{1,0} \to 0$, such that $1 = S_0 + V_0$ and the threshold $h^{\text{HIT}} = V_0$ such that the number of new infected individuals does not reach an extremum (but is monotonically decreasing). We then obtain the limiting case $\sigma_1 (1 - h^{\text{HIT}}) + \zeta \sigma_2 h^{\text{HIT}} = 1$, leading to

$$h^{\text{HIT}} = \frac{\sigma_1 - 1}{\sigma_1 - \zeta \sigma_2}\,, \tag{E16}$$

which indeed fits with the numerical plots in the right panel of Figure F1. Notice that for $\zeta \sigma_2 > 1$, we formally find $h^{\text{HIT}} > 1$: in this case, the efficacy of the vaccine is too low and herd immunity cannot be achieved. Furthermore, in the limit $\zeta \to 0$ (*i.e.* a perfect vaccine), eq. (E16) reduces to the usual definition (see *e.g.*[1]) $\lim_{\zeta \to 0} h^{\text{HIT}} = 1 - \frac{1}{\sigma_1}$.

S2 SIIRV with Time-Dependent σ_1 and Constant ε

In this appendix we explore another approach to implementing time-dependent rates into the

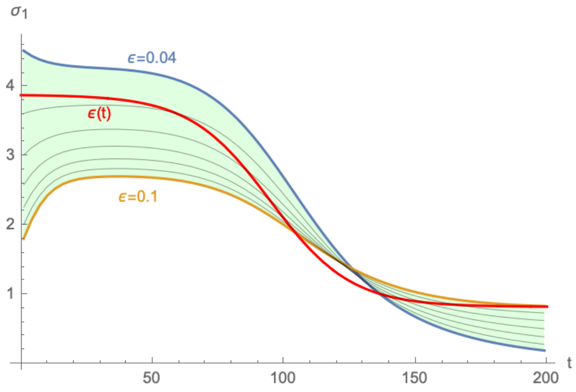

Figure F2. Time dependence of the infection rate σ_1 (left panel) for constant $\varepsilon \in [0.04, 0.1]$ that is needed to reproduce $I_c(t)$ of the form (9) with the compartmental model (1). The red curve shows as comparison the σ_1 obtained assuming a time-dependent ε as in Figure 2, using the same remaining parameters.

SIIRV model (1) and compare their impact on the VT-HP and V-HP models (4) and (4) respectively. Indeed, we have allowed both (σ_1, ε) to depend on time (leading to (11)). This has required us to assume the number of infectious individuals (10) associated with (9), which indeed correctly captured the numbers found in Germany and Austria (see Figures F12 and (F17) respectively). Similarly the time dependent (σ_1, ε) were similar to the results obtained in[2] in the context of a simpler compartmental model.

In this appendix, we shall rather make the assumption that ε is a constant in time.This assumes that (at least at short times) the recovery rate from the disease as well as the rate at which infected individuals can be found and isolated, does not change. In this case, a single equation for σ_1 is required, such that (9) is sufficient input to determine the latter as a function of time. Since in this case ε is a constant free parameter, we consider $\varepsilon \in [0.04, 0.1]$, which is roughly the range $\varepsilon(t)$ covered in the right panel of Figure 2. The result, in comparison to σ_1 obtained assuming a time-dependent ε are shown in Figure F3.

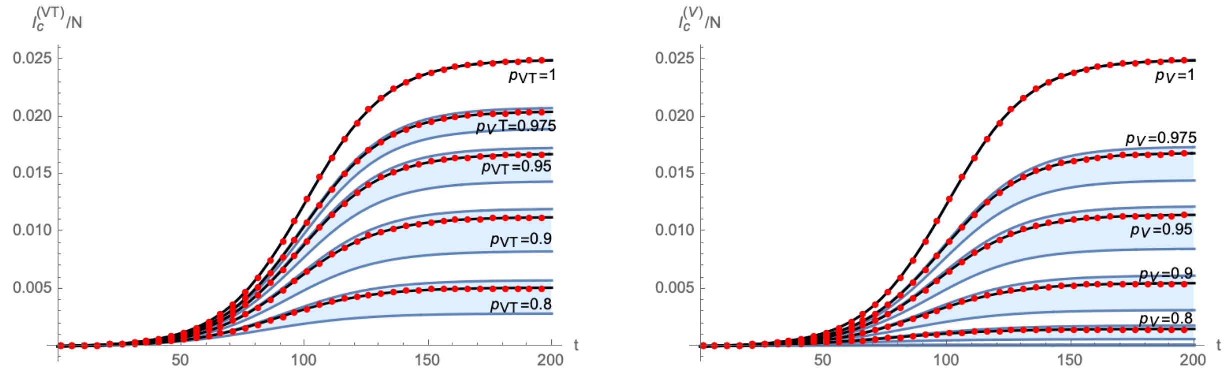

Figure F3. I_c as a function of time for different values of p using the VT-HP model (4) (left panel) or the V-HP model (6) (right panel). The blue bans correspond to the choice of $\varepsilon \in [0.04, 0.1]$, while the red dots give the result of the computation assuming time-dependent ε , along with their interpolation using a logistic function. Both plots use $A = 0.025$, $\lambda = 0.06$, $t_0 = 100$, $\delta = 14$, $\rho = 0.0003$, $\zeta = 0.1$, $\sigma_2/\sigma_1 = 1$ and $V_0 = 0.5$. The interpolating black lines correspond to approximations with logistic functions.

Using this time-dependence to implement the HPmodels (4) and (6) leads to different cumulative numbers of infected individuals as a function of time for different values of p_{V} and p_{VT}, as is shown in Figure F3, once more in comparison to the result of the main text which assumed a time-dependent ε. The blue bands in this plot represent the range obtained from $\varepsilon \in [0.04, 0.1]$. Notice, that by design all choices of ε reproduce the same function for $p = 1$. Furthermore, for all choices of ε

and p, I_c/N can be approximated by a logistic functions. We note that these plots reveal a qualitatively similar picture to the computations in the main part of the paper.

S3 Asymptotic Expansion for the HP-SIR Model

To get an intuition about the asymptotic behaviour of the HP-models (4) and (6), we consider as a simpler model the following two SIR models with a HP

$$\frac{dS}{dt} = -p_{\text{VT}}\,\gamma S I\,, \qquad \frac{dI}{dt} = p_{\text{VT}}\,\gamma S I - \varepsilon I\,, \qquad \frac{dR}{dt} = \varepsilon I\,, \qquad \text{with} \qquad \begin{array}{l} S(t=0)=S_0\,, \\ I(t=0)=I_0\,, \\ R(t=0)=0\,, \end{array} \tag{E17}$$

and

$$\frac{dS}{dt} = -p_{\text{V}}^2\,\gamma S I\,, \qquad \frac{dI}{dt} = p_{\text{V}}^2\,\gamma S I - \varepsilon I\,, \qquad \frac{dR}{dt} = \varepsilon I\,, \qquad \text{with} \qquad \begin{array}{l} S(t=0)=S_0\,, \\ I(t=0)=I_0\,, \\ R(t=0)=0\,, \end{array} \tag{E18}$$

with constant (γ,ε). These correspond to the usual SIR model (in the absence of vaccinations) in which the infection rate has been rescaled by some power of a parameter $p_{\text{V}}, p_{\text{VT}} \in [0,1]$. Although mathematically this is a rather trivial rescaling, we can learn certain qualitative features of the different power of the parameter p, which we shall also observe in the more complicated models in the main text of this paper that also accommodate vaccinations. Indeed, in absence of a vaccination dynamics, the asymptotic cumulative number of infected for the systems (E18) can be computed analytically

$$\frac{I_{\text{c}}^{(\text{VT})}(\infty,p_{\text{VT}})}{N} = 1 + \frac{W\left(-S_0\,\sigma\,p_{\text{VT}}\,e^{-\sigma p_{\text{VT}}}\right)}{\sigma p_{\text{VT}}}\,, \qquad \text{and} \qquad \frac{I_{\text{c}}^{(\text{V})}(\infty,p_{\text{V}})}{N} = 1 + \frac{W\left(-S_0\,\sigma\,p_{\text{V}}^2\,e^{-\sigma p_{\text{V}}^2}\right)}{\sigma p_{\text{V}}^2}\,, \tag{E19}$$

with $\sigma = \frac{\gamma}{\varepsilon}$ and where W is the Lambert function. $I_{\text{c}}^{(\text{VT,V})}(\infty,p)$ as a function of $p_{\text{VT,V}}$ (and normalised to $p=1$) is shown in Figure F4. As can be seen, for values of $p_{1,3}$ close to 1, the normalised asymptotic number of infected can be approximated by an exponential function of the form

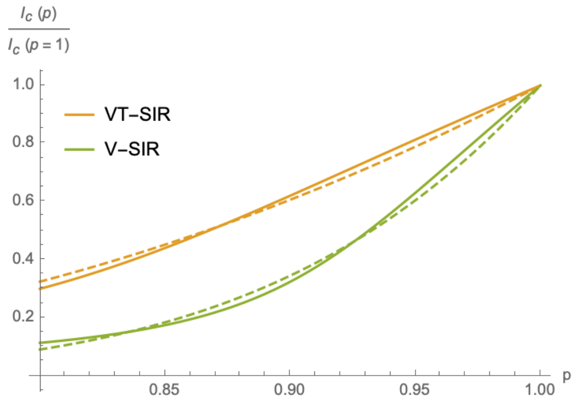

Figure F4. Asymptotic cumulative number of infected individuals for the HP-SIR models (E17) and (E18) as a function of $p_{\text{VT,V}}$ (indicated as p) and normalised to $p_{\text{VT}} = 1 = p_{\text{V}}$. The solid orange curve is the asymptotic solution of the model (E17) and the solid green curve the solution of the model (E18). The dashed lines are approximations of the solutions according to (E20) (with $\theta = 9.56$ for (E17) and $\theta = 4.49$ for (E18)). The plots use $S_0 = 0.99$ and $\sigma = 1.2$.

$$\frac{I_{\text{c}}(\infty,p)}{I_{\text{c}}(\infty,p=1)} \sim \exp\left(\theta\,\frac{p-1}{p}\right)\,, \qquad \text{with} \qquad \theta \in \mathbb{R}\,, \tag{E20}$$

for a constant θ that depends on S_0 and σ. In Figure F5 the quadratic error of the approximation $\mathscr{E}$

$$\Delta_{\mathscr{E}}^{(\text{VT,V})} = \sqrt{\int_{p_0}^{1} dp\,\left[\frac{I_{\text{c}}^{(\text{VT,V})}(\infty,p)}{I_{\text{c}}^{(\text{VT,V})}(\infty,1)} - \mathscr{E}\right]^2}\,, \tag{E21}$$

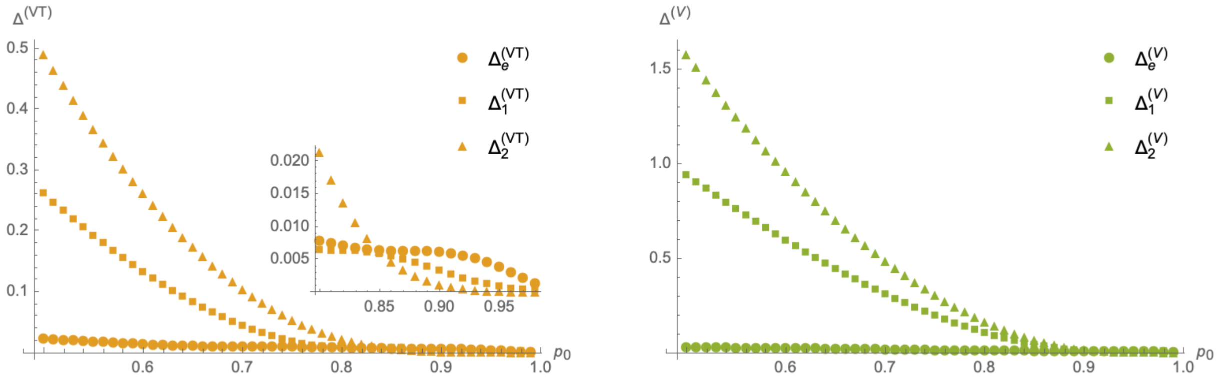

Figure F5. Comparison of the quadratic error (E21) for the approximation (E20) ($\Delta_e^{(\mathrm{VT,V})}$, indicated by the circles) with the error for the first order ($\Delta_1^{(\mathrm{VT,V})}$, indicated by the squares) and second order ($\Delta_1^{(\mathrm{VT,V})}$, indicated by the triangles) Taylor series expansion around $p = 1$ as a function of p_0. The left panel shows the estimation for the model (E17) and the right panel for (E18). Both plots use $S_0 = 0.99$ and $\sigma = 1.2$.

as a function of p_0 is compared for $\mathscr{E}$ given in (E20) with the first and second order of a Taylor series expansion around $p = 1$.

For the model (E18) a heuristic explanation of the approximation (E20) can be given as follows: let A be the total number of infectious contacts throughout the entire time duration of the pandemic, such that for small initial conditions $A \sim I_c^{(\mathrm{V})}(\infty)$. Considering these contacts for a value given value of p close to one and changing it by a small δp, leads to a modification of A that is (for small values of p) proportional to the number of infectious contacts for $p = 1$, which is roughly $A(p)/p^2$. Thus we find that A has to satisfy the approximate differential equation $\frac{dA}{dp} = \frac{\theta}{p^2} A(p)$ for some constant θ, whose solution is indeed (E20). For the model (E17), the same argument would lead to the modified differential equation $\frac{dA}{dp} = \frac{\theta}{p} A(p)$ which would suggest a linear behaviour. The detail in the left panel of Figure F5 shows more details of the approximation for p_0 in the vicinity of 1, in which case the linear Taylor series is a better approximation than (E20). However, for p_0 further from $p = 1$ the other dynamic of the system becomes more important and the heuristic argument is no longer justified.

S4 Vaccination Dynamics in the eRG Approach

To encode the vaccination dynamics in the eRG approach, we first give more details on implementing the presence of vaccinated individuals (represented by V_0 in the SIIRV compartmental model). As explained in the main text, this requires to allow for a dependence of the time-dependent $(\sigma_1(t), \varepsilon(t))$ on V_0. While ε shows only a negligible dependence on V_0, σ_1 for different values of V_0 are shown in the left panel of Figure F6. This dependence can also be translated in a V_0 dependence of $(A_\sigma, \lambda_\sigma, t_\sigma, \delta_\sigma)$ in eq.(11): as shown in the right panel of Figure F6, the parameter A_σ depends linearly on V_0, while δ_σ (and also λ_σ and t_σ) to first approximation are independent of V_0. We remark that the linear dependence of A_σ is compatible with results in[3] where a simple lattice model of susceptible, infectious, removed and vaccinated sites was considered which showed a linear relation between the asymptotic number of infected individuals and the initial number of vaccinated lattice sites, up to the herd immunity threshold.Indeed, although the paper[3] never explicitly discusses herd immunity, it reports a numerical instability of the lattice simulation starting at $V_0 \sim 25\%$ of lattice sites. With the data provided for the plot in[3], this roughly seems compatible with a phase transition at $h^{\mathrm{HIT}} = 1 - \frac{\varepsilon_*}{\gamma_*} = 1/3$, which would indeed manifest itself via a numerical instability in this approach. The impact of a HP-model (characterised by an efficacy parameter p) as a function of the individuals vaccinated before the outbreak of the wave (characterised by V_0) can be captured by plotting the asymptotic cumulative number of infected individuals in the (p, V_0) plane, as is schematically shown for a VT-HP- and V-HP-model in the left panel of Figure F7.

Furthermore, in[3] a proposal was made how to include a non-trivial vaccination rate ρ in the eRG approach. The approach is based on replacing the parameter A_0 of the β-function (8) by a time dependent function $A_0(t)$. For any given t, the change in the asymptotic number of infected individuals can only be a function of $A_0(t) - I_c(t)$, thus suggesting the differential equation $\frac{dA_0}{dt} = f(A_0(t) - I_c(t))$, for a continuous function $f : \mathbb{R}_+ \to \mathbb{R}_+$. The latter was assumed to be linear in[3] (in fact $f(x) = -\rho x$). An immediate generalisation, which allows us to make contact with the time-dependent SIIRV model is the simple modification given in the second equation of (13). Indeed, numerical solutions for different choices of w are shown in the right panel of Figure F7: while (for the parameters (A_0, λ_0, t_0) at $t = 0$ chosen in this plot), $w = 1$ leads to a qualitatively different function, $w = 0.725$ leads to an acceptable agreement. Furthermore, Figure F7 shows the impact of the initial number of vaccinated individuals on the asymptotic cumulative number of infected individuals, which can be approximated by a function of the form

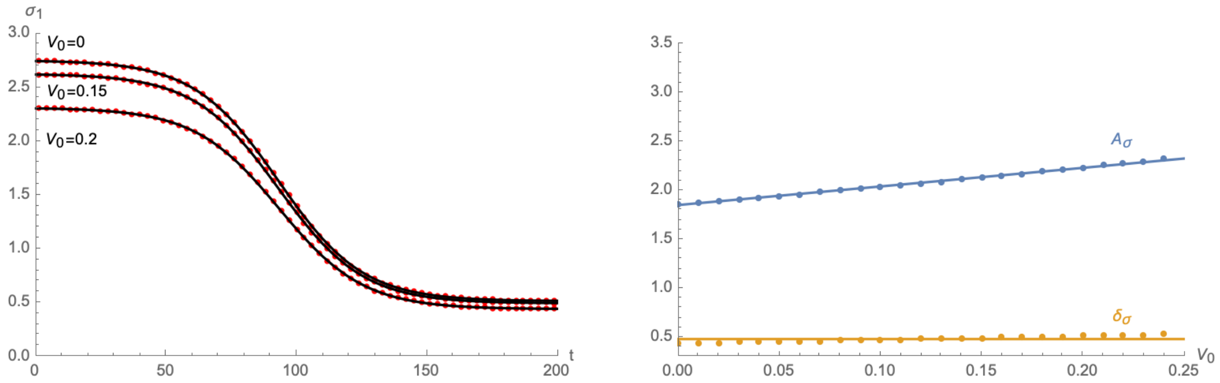

Figure F6. Left panel: time dependence of σ_1 for different values of V_0. Right panel: A_σ and δ_σ as functions of V_0 required to reproduce the result from the compartmental model (1). Both plots use $A_0 = 0.025$, $\lambda_0 = 0.06$, $t_0 = 100$, $c = 14$, $\rho = 0$, $\zeta = 0$ and $\sigma_2/\sigma_1 = 1$.

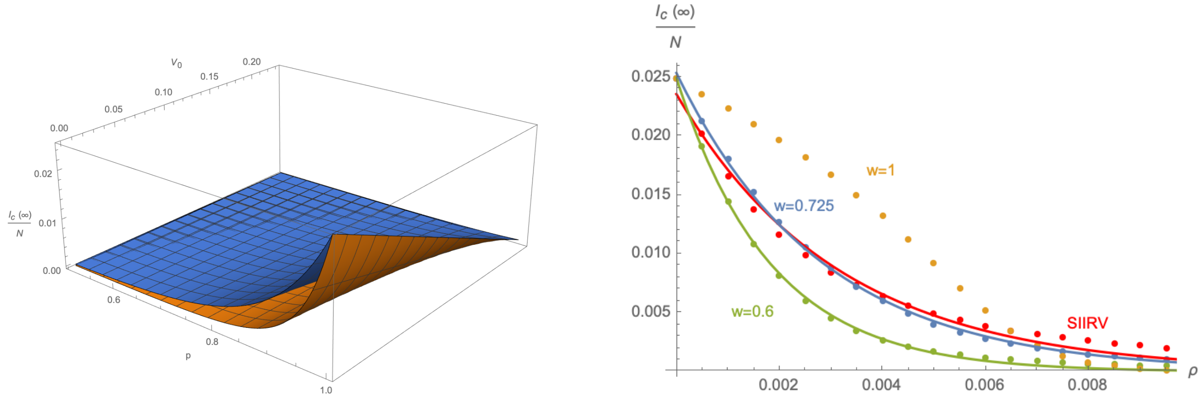

Figure F7. Left panel: Plot of $I_c(\infty)/N$ as a function of (p, V_0). The blue surface refers to a VT-HP-model, while the orange one refers to a V-HP-model. The plots use $\rho = 0.0008$, $\zeta = 0.2$ and $\sigma_2 = \sigma_1$ as well as $A_0(p = 1) = 0.025$, $t_0(p = 1) = 100$ and $\lambda_0(p = 1) = 0.06$. Right panel: Comparison of the impact of the vaccination rate on the cumulative number of infected individuals predicted by the SIIRV model (1) (with time dependent (σ_1, ε), red curve) to the eRG model using eq. (13) for different values of w. The straight lines correspond to interpolations of the form $a\,e^{-b\rho}$ for suitable numerical coefficients a, b. The plot uses $A_0 = 0.025$, $\lambda_0 = 0.06$, $t_0 = 100$, $V_0 = 0$ and $\zeta = 0.2$.

$a e^{-bV_0}$ for some suitable constants (a, b).

S5 Equivalence of HP-Models

In Figure 5 we have shown plotted equivalent values for the efficacy parameters p_{VT} and p_{V} of a VT-HP and V-HP model that lead to the same asymptotic cumulative number of infected individuals. This relation assumed, however, that all remaining parameters of the two HP-models, in particular the removal rate ε remain the same. Since a V-HP-model offers less incentive for individuals to get tested (unless they present clear symptoms), this is not a fully justified assumption, since the testing rate in an important part in quarantining and removing infectious individuals.

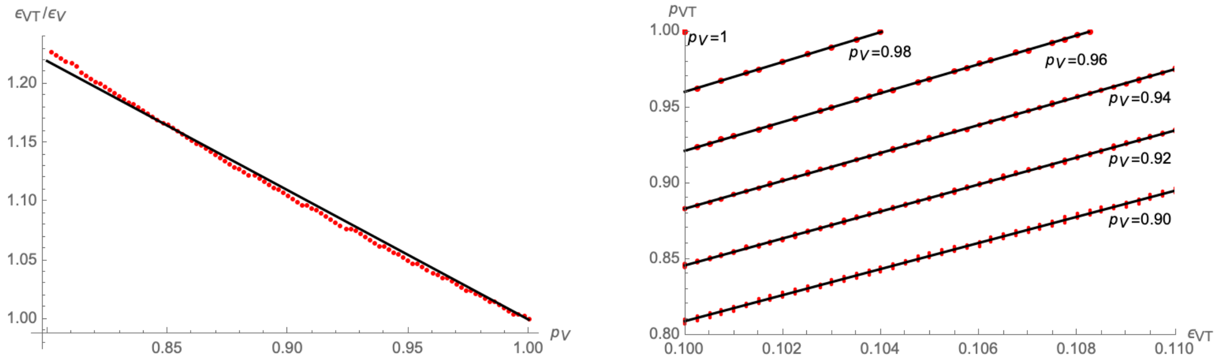

Figure F8. Value of $\varepsilon_{\mathrm{VT}}/\varepsilon_{\mathrm{V}}$ that is necessary for given $p_{\mathrm{V}} = p_{\mathrm{VT}}$ to lead to the same asymptotics of (4) and (6).

In Figure F8 we have therefore plotted the relation of (constant) $\varepsilon_{\mathrm{VT}}$ in the compartmental SIIRV models (4) (normalised to $\varepsilon_{\mathrm{VT}}$ in (6)) that is necessary for given $p_{\mathrm{V}} = p_{\mathrm{VT}}$ to lead to the same asymptotics of (4) and (6). We remark, in particular, that the right panel of Figure F8 suggests a linear equivalence between the parameter p_{VT} and the removal rate ε. This can be explained by the fact that in the model (4) the asymptotic cumulative number of infected individuals depends to good approximation on the rates only in the combination $\sigma_1 = \frac{\gamma_1}{\varepsilon} = \sigma_2$. Furthermore, for the model (4), the parameter p_{VT} can be reabsorbed by a rescaling of γ_1. This explains why a linear relation among p_{VT} and ε yields equivalent asymptotic results. These results can be extended to the eRG approach (or equivalently the compartmental models with time-dependent rates): first of all the equivalence relation between p_{VT} and p_{V} is shown in the left panel of Figure F9, which is virtually the same as in the case of the SIIRV model with constant rates as in the left panel of Figure 5. In order to allow for a different removal rate, we have more possibilities to adapt the time-dependent function $\varepsilon_V(t)$ in (11): the simplest option is to consider a time independent shift $\delta_\varepsilon \to \delta_\varepsilon + \Delta_\varepsilon$ with a constant parameter Δ_ε. Comparing numerical computations of different values of $(p_{\mathrm{V}}, \Delta_{\varepsilon_1})$ leads to equivalences as shown in the right panel of Figure F9.

S6 Examples

S6.1 HP in Different European Countries

In this appendix we provide a short overview over different HP that have been discussed or introduced in some European countries. Our focus is on Austria, Denmark, France and Italy, which we have studied as examples in this paper.

- **EU Digital COVID Certificate:** The EUDCC (also called Green Pass) was introduced in the European Union on 01/07 with the goal to facilitate travel within its 27 member states as well as Switzerland, Iceland, Norway, and Liechtenstein[4]. It is a VT-HP and is issued to individuals, who have been vaccinated, have recently tested negative for SARS-Cov-2 (negative PCR test) or have recently recovered from a COVID-19 infection. It exempts individuals from further testing or quarantine when traveling within the participating countries.

- **Austria**: Austria has introduced a VT-HP model as early as 19/05, whose range of applicability has has since been changed several times: following the '3-G-Regel' (geimpft, getestet, genesen)[5] unrestricted access to public life is granted to individuals only who are either vaccinated, have recently tested negative for SARS-Cov-2 (PCR test not older than 72 hours or antigen test not older than 48 hours) or have recovered from a previous COVID-19 infection (medical attestation not older than 6 months or a test for antibodies not older than 3 months). Restrictions in particular apply to establishments of the tourism industry, gastronomy and the attendance of public events. A plan unveiled by the government on 08/09 provides further restrictions to take effect as a function of the number of hospitalised individuals.

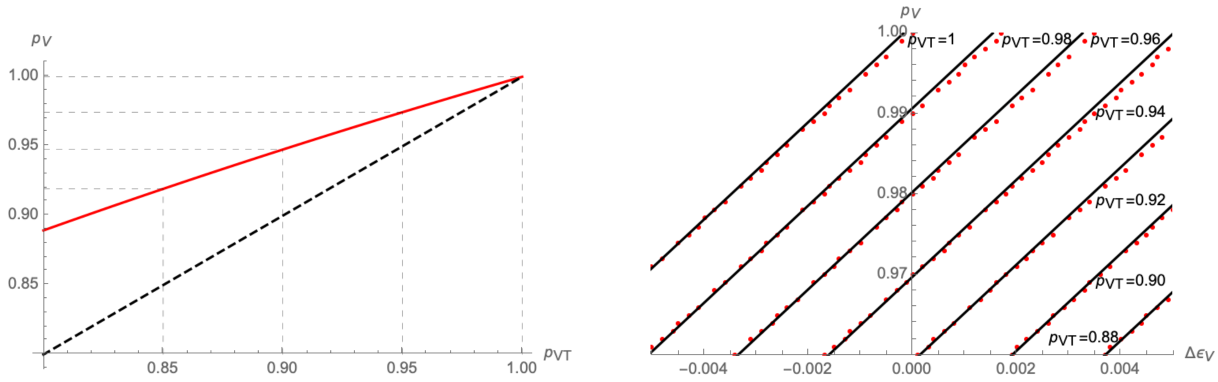

Figure F9. Imposing the same asymptotic cumulative number of infected individuals leads to implicit relations between the parameters of the Green Pass models (4) and (6). Left panel: assuming all other parameters of the models to remain the same entails a linear equivalence relation between the efficacy $(p_{\mathrm{V}}, p_{\mathrm{VT}})$ of the models (4) and (6). Right panel: assuming the change $\delta_\varepsilon \to \delta_\varepsilon + \Delta_\varepsilon$ in the time-dependent parameter $\varepsilon_{\mathrm{V}}(t)$ leads to a degeneracy of the parameters that lead to the same asymptotic cumulative number of infected individuals for the model (6): all combinations of parameters along the (interpolating) solid lines are 'equivalent' to model (4) with the indicated value of p_{VT}. The plots use $A_0 = 0.025$, $\lambda_0 = 0.06$ (at $t = 0$), $t_0 = 100$, $V_0 = 0.3$ and $\zeta = 0.15$.

- **Denmark:** In Denmark, a coronapas (or corona-passport), a VT-HP has been introduced in the end of April[6], which is required for Health to nightclubs and discotheques, as well as public events. Its applicability has changed since then and is currently for example no longer required for visiting restaurants and cafés. Individuals eligible for a corona-passport need to either prove that they have been vaccinated, have previously recovered from COVID-19, or have recently tested negative for SARS-Cov-2 (PCR test not older than 96 hours or rapid antigen test not older than 72 hours).
- **France:** In France a 'pass sanitaire' was introduced on 01/06, first for events with more than 1000 participants, which was later gradually extended to cover mover and more aspects of public life[7]. Currently, individuals can obtain a 'pass sanitaire' if they have been completely vaccinated, have tested negative for SARS-Cov-2 (PCR test not older than 72 hours or a supervised self-test), or have recently recovered from COVID-19 (positive test not older than 6 months).
- **Italy:** In Italy, since 06/08, Health to public places and cultural events (stadiums, museums, theaters, cinemas, *etc.*) as well as sport centers (swimming pools and gyms) requires a 'green pass'[8], which is an extension of the EU's digital COVID certificate. Currently, the pass is not required for long distance travel across the country.

S6.2 Germany

In this section we present additional information and results concerning the introduction of HP-models in Germany. We first start with presenting more detailed epidemiological data: since the outbreak of the COVID-19 pandemic, Germany has been hit by three waves, which can be seen from the cumulative number of infected individuals as plotted in Figure F10. We have fitted each wave individually by a logistic function

$$I_{\mathrm{c}}^{\mathrm{wave}}(t) = I_{\mathrm{c},0} + \frac{A_0}{1 + e^{-\lambda_0 (t - t_0)}}\,, \tag{E22}$$

where the parameters for each wave are given in the following table:

**wave**	A_0	λ_0	t_0	$I_{\mathrm{c},0}$
wave 1	177112 ± 918	0.119 ± 0.003	50.2 ± 0.3	0
wave 2	$2.33692 \cdot 10^6 \pm 10080$	0.0395 ± 0.0003	303.4 ± 0.2	190913 ± 3631
wave 3	$1.38258 \cdot 10^6 \pm 10061$	0.0595 ± 0.0008	424.3 ± 0.2	$2.37996 \cdot 10^6 \pm 7943$

The cumulative number of fully immunised individuals is shown in Figure F11 (Here we also follow a recommendation[10] of the Robert Koch Institut (RKI) and count an individuals as fully immunised 14 days after having received the last dose of a full vaccination scheme), which can be approximated by a piecewise linear function: indeed, a strong increase in the vaccination

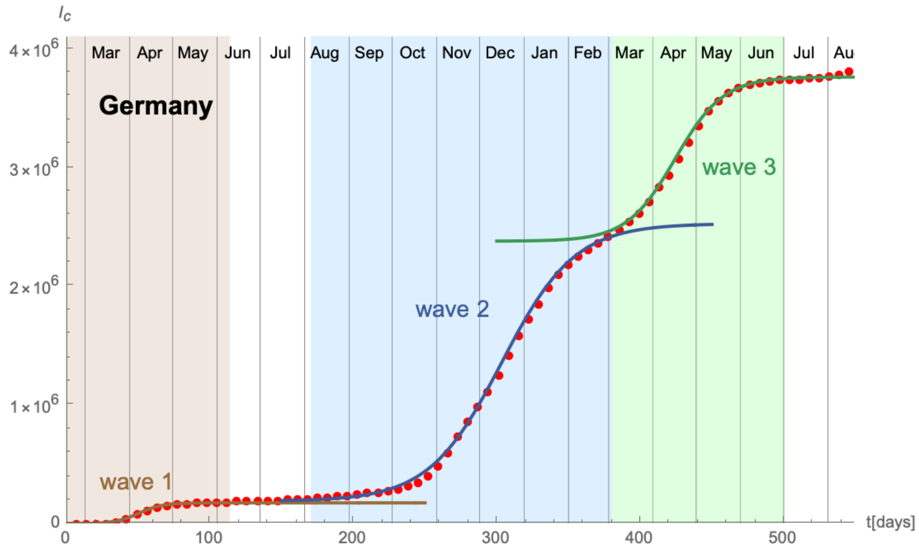

Figure F10. Fitting the first three waves in Germany. The red dots represent the cumulative number of infected individuals as reported in[9]. The coloured regions indicate the time frames used to fit the three different waves, whose fits are shown by the solid lines. The fit parameters are exhibited in the table in the text.

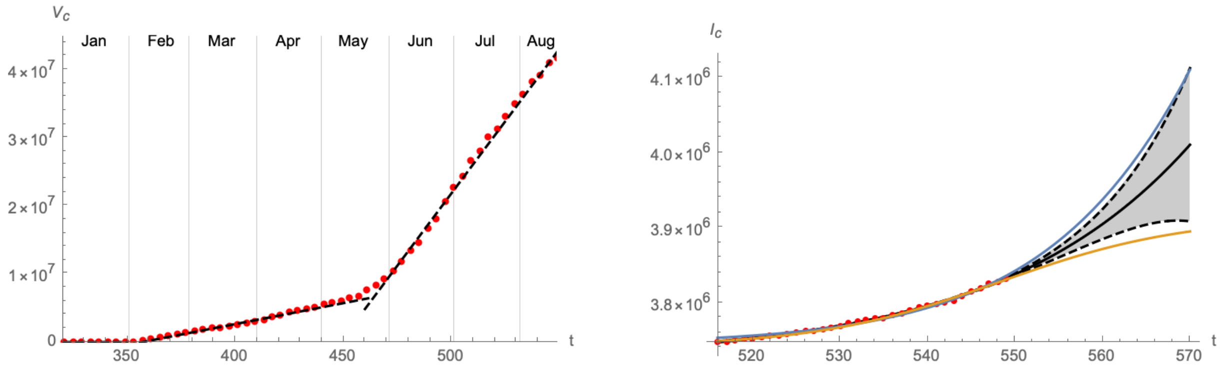

Figure F11. Left panel: Cumulative number of fully immunised individuals in Germany as a function of time. Right panel: the cumulative number of cases suggests the onset of a fourth wave in Germany in the late summer/beginning of fall. The gray region indicates the 90% confidence interval of the fit of the reported cases with a logistic function, with the blue and orange curve representing an approximation of the extremal cases through logistic functions.

rate can be seen in the middle of May 2021. Finally, analysing the data of the month of July 2021 suggests the onset of a fourth wave. Fitting of these data with a logistic function is shown in the right panel of Figure F10, which, however, also indicates the 90%-confidence interval for the development of the following 3 weeks. The blue and the orange curve are the extreme cases approximated by logistic functions with the parameters given by eq. (18) in the main body of this paper, respectively

Focusing on waves 2 and 3, using (10), we compute the number of (active) infectious individuals associate with the cumulative number of infected individuals. As shown in Figure F12, comparing with the active cases reported in[9] the best fit is achieved using $\tau = 17$ days. Based on the functions $I(t)$ and I_c, we next derive time-dependent parameters $(\sigma_1(t), \varepsilon(t))$ such that the SIIRV model (1) reproduces the cumulative number of infected individuals. For both waves, we find that the rates can be approximated by logistic functions of the form given in eq. (11), where the fitting parameters $(A_{\sigma,\varepsilon}, \lambda_{\sigma,\varepsilon}, \tau_{\sigma,\varepsilon}, \delta_{\sigma,\varepsilon})$ are shown in the following table:

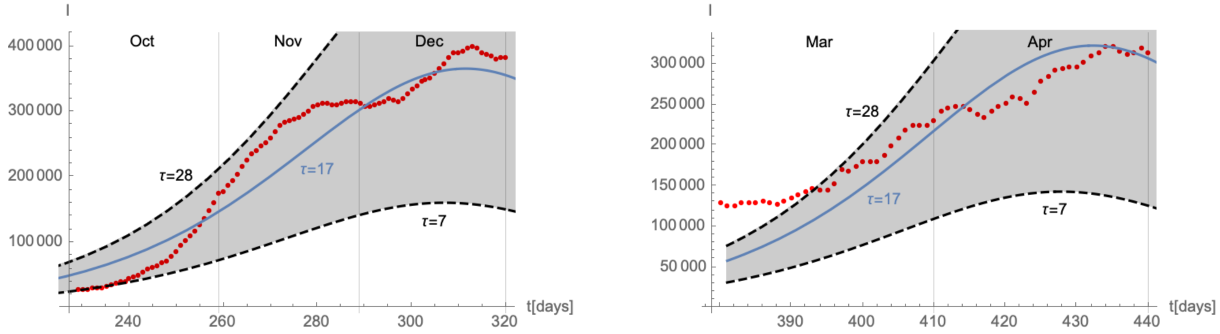

Figure F12. Fitting of the (active) number of infectious individuals derived from the cumulative number of infected individuals (black band) compared to the reported cases (red dots) in Germany. The approximation shows the range of the parameter $\tau \in [7, 28]$, with the best fit indicated by the blue line for $\tau = 17$.

	$A_{\sigma,\varepsilon}$		$\lambda_{\sigma,\varepsilon}$		$t_{\sigma,\varepsilon}$		$\delta_{\sigma,\varepsilon}$	
	wave 2	wave 3	wave 2	wave 3	wave 2	wave 3	wave 2	wave 3
σ_1	1.333	2.156	0.0403	0.064	124.8	36.9	0.547	0.43
ε	0.037	0.056	0.0395	0.0595	147.5	58.3	0.046	0.039

The functions (σ_1, ε) are also plotted in Figure F13. During wave 2, vaccines were not available, such that the above fitting uses $\zeta = 0 = \rho$, while for wave 3 we have used $\zeta = 0.15$ as well as $\rho = 0.0008$. In Figure F14, the cumulative number of fully vaccinated individuals is compared with the actual number reported by the RKI[10]: while the agreement is excellent for the months March and April, the vaccination rate has been significantly increased in Germany in the middle of May. Since this occurred towards the end of wave three (*i.e.* after the maximum in the number of active infectious individuals has been reached, as can be seen in the right panel of Figure F12), we have ignored this effect and have fitted the entire wave with $\rho = 0.0008$.

Finally, for the impending wave 4, Figure F15 shows the impact of different combinations of the efficacy parameters of the HP-models and the number of vaccinated individuals on the asymptotic cumulative number of infected individuals at the end of the wave in the form of a plot in the (p, V_0) plane.

S6.3 Austria

We next also present additional information and results concerning the introduction of HP-models in Austria. As Germany, Austria has also been hit by 3 waves since the beginning of the pandemic. As is showcased in Figure F16 each of these can be fit with a logistic function of the type (E22) with the following parameters

**wave**	A_0	λ_0	t_0	$I_{c,0}$
wave 1	15361 ± 70	0.182 ± 0.004	43.0 ± 0.2	0
wave 2	331987 ± 4907	0.0785 ± 0.002	274.0 ± 0.3	25832.6 ± 3512
wave 3	253295 ± 1031	0.0491 ± 0.0003	405.6 ± 0.1	402220 ± 722

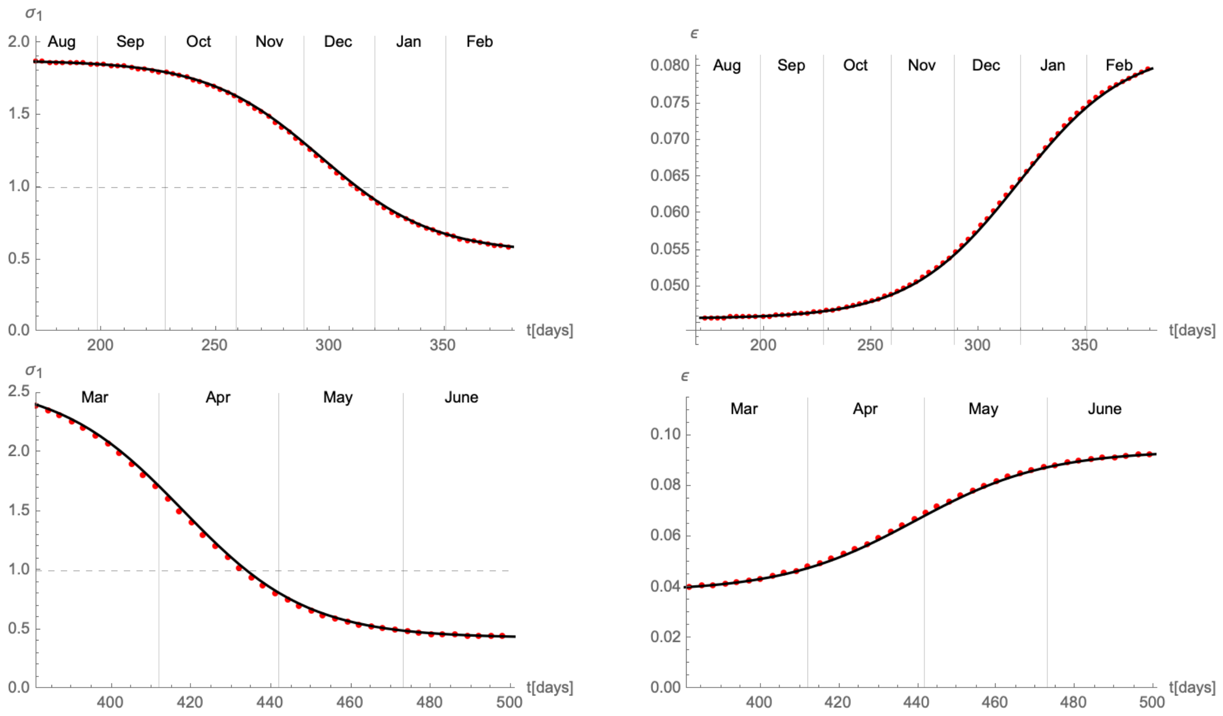

Figure F13. The time-dependent parameters (σ_1, ε) needed to match the compartmental model (1) (red dots) along with their interpolations (black lines) following (11). Top panels: wave 2, bottom panels: wave 3. In the latter case we have used $\zeta = 0.15$ as well as $\varepsilon = 0.0008$.

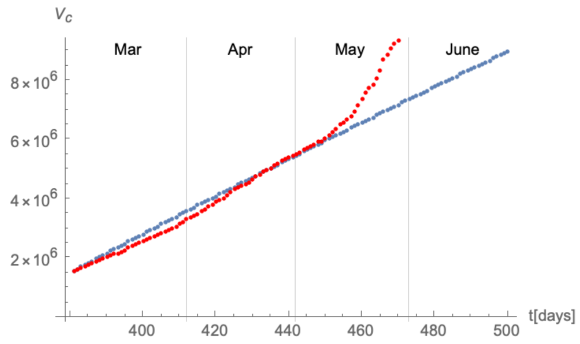

Figure F14. Comparison of cumulative number of fully vaccinated individuals reported by the RKI[10] (red) with the numerical calculation of the SIIRV model (blue).

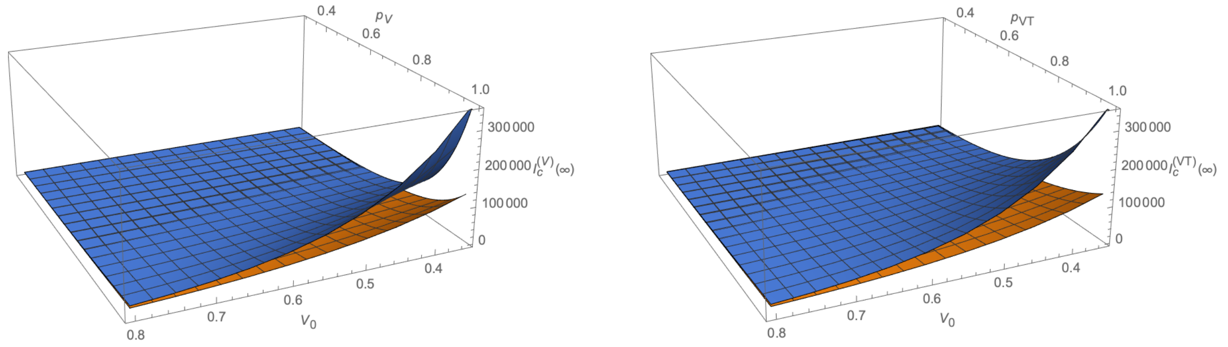

Figure F15. Extremal predictions of I_c in the plane of (p, V_0), assuming $\rho = 0.008$, $\zeta = 0.15$. The left panel shows the plot for a V-HP, while the right panel for a VT-HP.

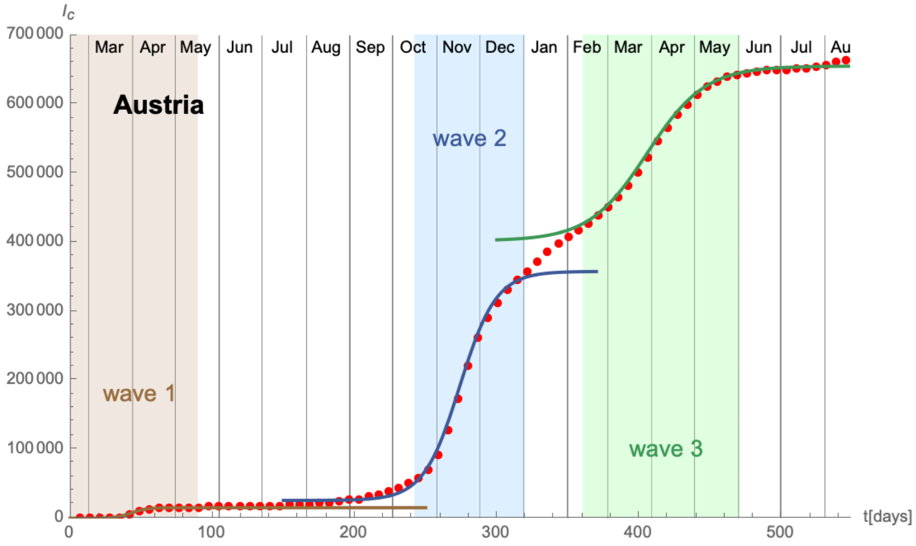

Figure F16. Fitting the first Three Waves in Austria. The red dots represent the cumulative number of infected individuals as reported on[9]. The coloured regions indicate the time frames used to fit the three different waves, whose fits are shown by the solid lines. The fit parameters are exhibited in the table in the text.

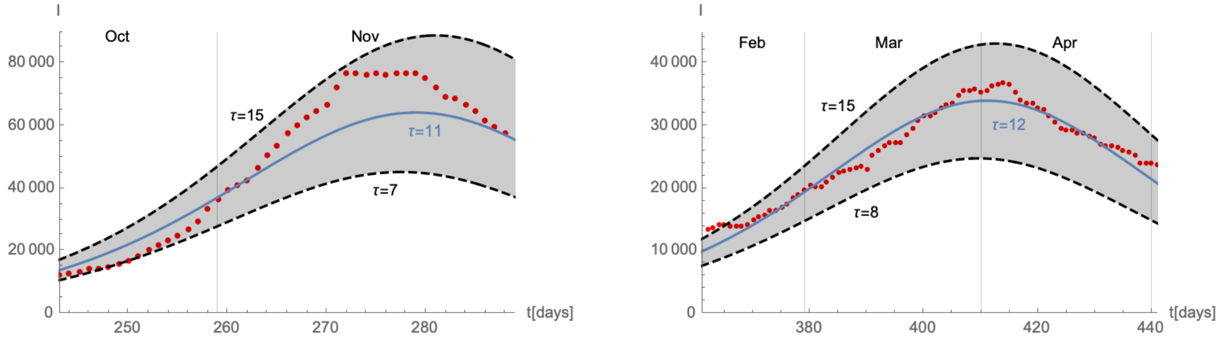

Figure F17. Fitting of the (active) number of infectious individuals derived from the cumulative number of infected individuals (black lines) compared to the reported cases (red dots) in Austria. The gray band represents different values for the parameter τ in (10) with the blue line giving the best numerical fit.

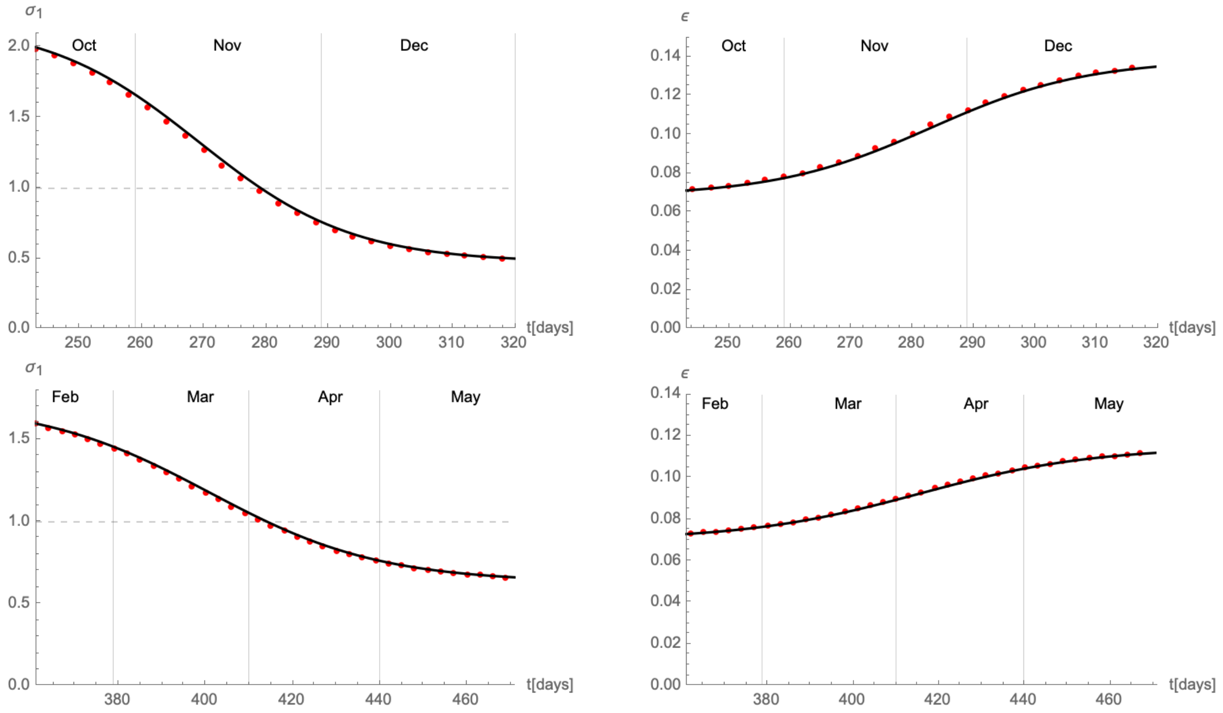

Figure F18. The time-dependent parameters (σ_1, ε) for the compartmental model (1) (red dots) along with their interpolations (black lines) following (11) needed to match the epidemiological data for the second (top panels) and third (bottom panels) waves in Austria.

The number of (active) infectious individuals derived for waves 2 and 3 from these data is shown in Figure F17 and compared to the cases reported on[9]. The grey band shows different approximations depending on the parameter τ, which in turn is a measure for how long on average an infected individual remains infectious. Similar to the situation in Germany (see Figure F12) good agreement can be achieved for $\tau \in [7, 15]$ with the best fit corresponding to $\tau = 11$ and $\tau = 12$ for waves 2 and 3 respectively. The time-dependent functions $(\sigma_1(t), \varepsilon(t))$ that are needed to fit the data of waves 2 and 3 (where no HP model was imposed) are shown in Figure F18.

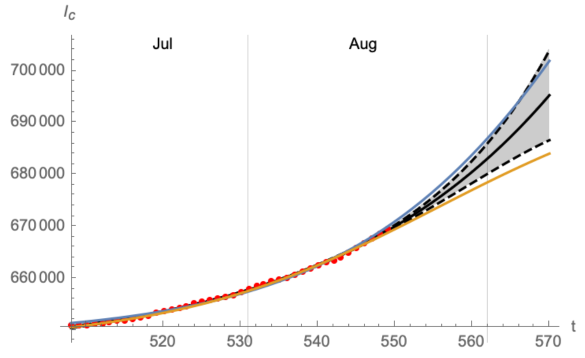

Figure F19. Cumulative number of infected in Austria starting from July/2021.

Finally, regarding a possible fourth wave in the end of summer/beginning of fall 2021, the infection numbers starting from 07/July are shown in Figure F19 along with a 90% confidence interval. The blue and orange curves correspond to fits of the

extreme cases with logistic functions with the parameters

$$A_0^+ = 200000\,, \qquad \lambda_0^+ = 0.053\,, \qquad t_0^+ = 589.1\,, \qquad \delta_0^+ = 648808\,,$$
$$A_0^- = 51856\,, \qquad \lambda_0^- = 0.059\,, \qquad t_0^- = 555.3\,, \qquad \delta_0^- = 647643\,. \tag{E23}$$

Figure F20 shows the impact of different combinations of the efficacy parameters of the V-HP-models and the number of vaccinated individuals on the asymptotic cumulative number of infected individuals at the end of the wave in the form of a plot in the $(p_{\rm V}, V_0)$ plane.

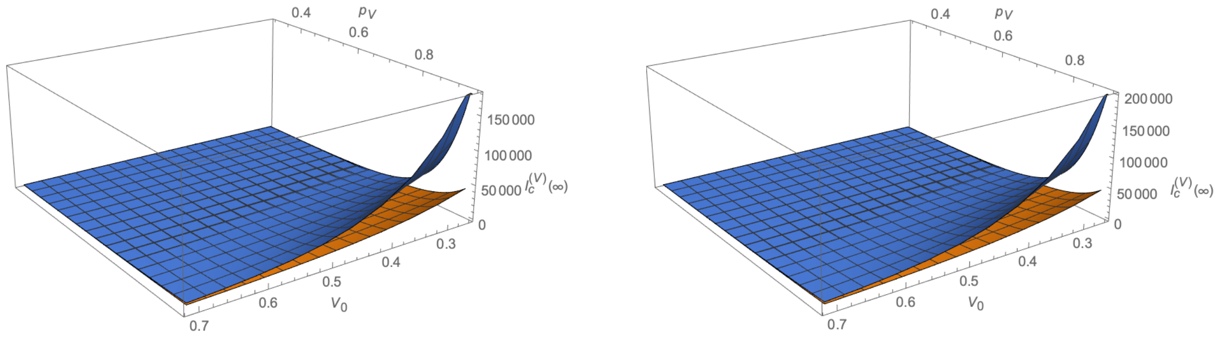

Figure F20. Asymptotic cumulative number of infected individuals as a function of p_V and V_0 for the fourth wave Austria. Both plots assume $\sigma_2/\sigma_1 = 1$, $\rho = 0.0085$ as well as $p_{\rm VT} = 0.9$ (left panel) or $p_{\rm VT} = 0.8$ (right panel) for the VT-HP currently in place in Austria.

S6.4 Further Examples

In the following we briefly show the results of a similar analysis as for Germany and Austria also for France, Denmark and Italy, which have similar VT-HP models in place as Austria. For more details on the concrete technical implementations of the VT-HP, see the previous Appendix.

France

As a further example we consider France, where since 09/08 a 'pass sanitaire' is obligatory, which follows roughly the VT-HP model (4). Since thus the situation is to some extent similar as in Austria, we focus our discussion on a potential fourth wave in the end of summer/beginning of fall. At the time when this paper was being finalised, the actual infection numbers in France have been dropping, indicating the end of this wave. While this assessment neglects possible new developments related to the opening of schools and universities in September, we can fit the data under this assumption rather precisely with a logistic function, as is shown by the gray band in Figure F21, which corresponds to the 90% confidence interval. We again consider the two cases that the current pass-sanitaire has an efficiency of $p_{\rm VT} = 0.8$ and $p_{\rm VT} = 0.9$ and compare the results with the stronger V-HP. Indeed repeating the same analysis as in the previous subsection yields the plots in Figures F21. The results again support an equivalence of the type (19) between the parameters $p_{\rm V}$ of the V-HP and $p_{\rm VT}$ of the VT-HP model.

Denmark

As a further example we consider Denmark, where a VT-HP (called Corona-Passport) of the form (4) has been imposed, which follows roughly the model (4). As in the case of France, we focus exclusively on the development in the summer 2021: the epidemiological data along of a fit with a logistic function are shown by the black line in Figure F22 (with the gray band denoting its 90% confidence interval). In absence of studies estimating the efficiency of the 'Corona Passport', we consider the two cases of $p_{\rm VT} = 0.8$ and $p_{\rm VT} = 0.9$ and compare the results with a stronger V-HP. Indeed repeating the same analysis as in the previous subsection yields the plots in Figures F22. The results again support an equivalence of the type (19) between the parameters $p_{\rm V}$ of the V-HP and $p_{\rm VT}$ of the VT-HP model.

Italy

As a final example we consider Italy, where a VT-HP of the form (4), has been imposed. As in the previous cases, we focus exclusively on the development in the summer 2021: the epidemiological data along of a fit with a logistic function are shown

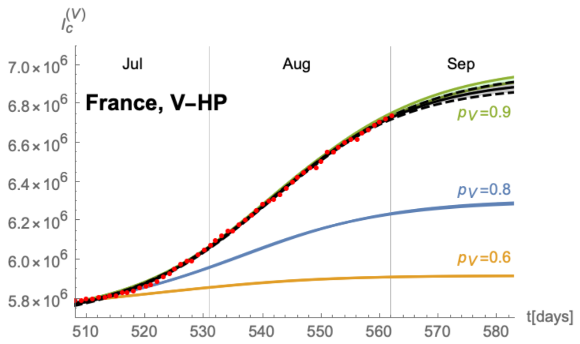

Figure F21. Predicted time evolution of the cumulative number of infected individuals for different values of the efficacy p_{V} of the V-HP model. Both plots assume $\sigma_2/\sigma_1 = 1$, as well as $p_{\mathrm{VT}} = 0.8$ (left panel) and $p_{\mathrm{VT}} = 0.9$ (right panel) for the VT-HP currently in place in Austria.

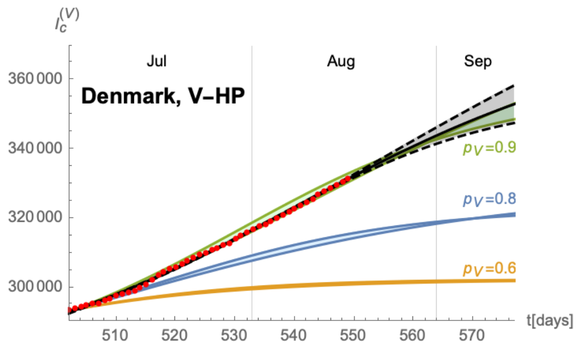

Figure F22. Predicted time evolution of the cumulative number of infected individuals for different values of the efficacy p_{V} of the V-HP model. Both plots assume $\sigma_2/\sigma_1 = 1$, as well as $p_{\mathrm{VT}} = 0.8$ (left panel) and $p_{\mathrm{VT}} = 0.9$ (right panel) for the VT-HP in Denmark.

as the black line in Figure F23 (with the gray band representing its 90% confidence interval). In absence of studies estimating the efficacy of the VT-HP currently in place in Italy, we consider the two cases of $p_{VT} = 0.8$ and $p_{VT} = 0.9$ and compare the results with a stronger V-HP. Indeed repeating the same analysis as in the previous subsection yields the plots in Figures F23. The results again support an equivalence of the type (19) between the parameters p_V of the V-HP and p_{VT} of the VT-HP model.

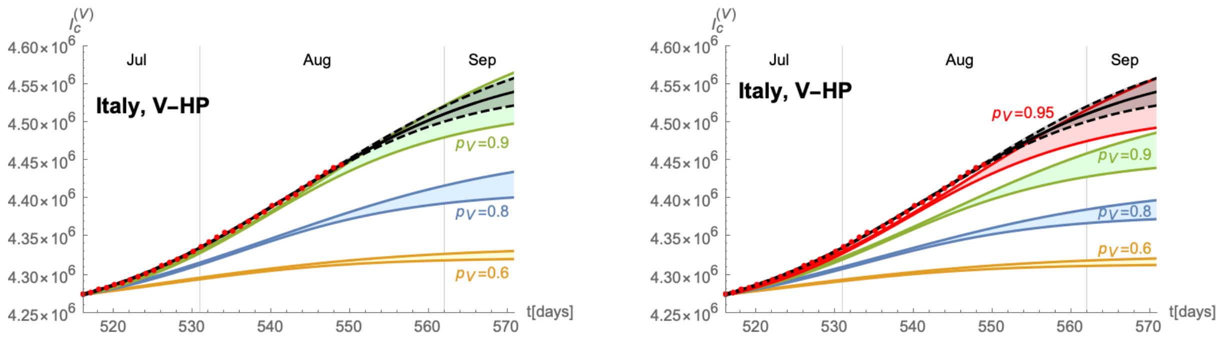

Figure F23. Predicted time evolution of the cumulative number of infected individuals for different values of the efficacy p_V of the V-HP model. Both plots assume $\sigma_2/\sigma_1 = 1$, as well as $p_{VT} = 0.8$ (left panel) and $p_{VT} = 0.9$ (right panel) for the VT-HP currently in place in Ital.

References

1. Garnett, G. P. Role of Herd Immunity in Determining the Effect of Vaccines against Sexually Transmitted Disease. *The J. Infect. Dis.* **191**, S97–S106, DOI: 10.1086/425271 (2005). https://academic.oup.com/jid/article-pdf/191/Supplement_1/S97/2760041/191-Supplement_1-S97.pdf.
2. Della Morte, M. & Sannino, F. Renormalisation Group approach to pandemics as a time-dependent SIR model. *Front. Phys.* **8**, 583, DOI: https://doi.org/10.3389/fphy.2020.591876 (2021).
3. Cacciapaglia, G., Cot, C., Islind, A. S., Óskarsdóttir, M. & Sannino, F. Impact of us vaccination strategy on covid-19 wave dynamics. *Sci. Reports* **11(1)**, 1–11 (2021). 2012.12004.
4. BBC. The eu vaccine 'passport' and what it means for travel. https://www.bbc.com/news/explainers-57665765 (2021).
5. Bundesministerium Soziales, P. u. K., Gesundheit. Coronavirus - aktuelle massnahmen. https://www.sozialministerium.at/Informationen-zum-Coronavirus/ (2021).
6. BBC. Coronapas: The passport helping denmark open up after covid. https://www.bbc.com/news/world-europe-56812293 (2021).
7. French Gouvernment. Informations coronavirus. https://www.gouvernement.fr/info-coronavirus (2021).
8. Guardian, T. Italy imposes 'green pass' restrictions on unvaccinated people. https://www.theguardian.com/world/2021/jul/22/italy-covid-19-green-pass-vaccinations-restrictions (2021).
9. Worldometer. Coronavirus cases. https://www.worldometers.info/coronavirus/ (2021).
10. Koch-Institut, R. Robert koch-institut. https://www.rki.de/EN/Home (2021).
